# Mechanisms of Engagement With Mobile Health Apps for Adults With Long-Term Conditions: Overview of Systematic Reviews

**DOI:** 10.2196/88382

**Published:** 2026-07-24

**Authors:** Jeni Baykoca, Goretti Hurtado Barbeito, Christina Joanne Pearce, Madison Milne-Ives, Joanna Hudson, Sam Norton, Rona Moss-Morris

**Affiliations:** 1 Department of Health Psychology, Institute of Psychiatry, Psychology and Neuroscience, King's College London, Guy's Hospital, Bermondsey Wing, 5th Floor, Health Psychology Section, London, England, SE1 9RT, United Kingdom, +44 7775485648

**Keywords:** mHealth, mobile apps, chronic disease, motivation, digital health, user-centered design, health behavior, self-management, telemedicine

## Abstract

**Background:**

Engagement is a necessary precondition for the effectiveness of mobile health (mHealth) apps for long-term physical health conditions (LTCs), particularly as health systems increasingly prioritize the deployment of scalable, self-guided digital interventions. Outside controlled research settings, where clinician involvement often drives engagement, little is known about whether, how, and why people engage with mHealth apps based on intrinsic motivation alone. Existing systematic reviews have cataloged behavioral engagement indicators but rarely assess the mechanisms underlying engagement.

**Objective:**

This overview of systematic reviews aimed to (1) synthesize evidence on how engagement with mHealth apps for LTCs is defined, measured, and associated with health outcomes, (2) explore intrinsic and extrinsic motivational processes underlying engagement, and (3) provide practical guidance for developing scalable, user-centered digital health interventions that sustain sufficient engagement with minimal reliance on external drivers. Uniquely, we interpreted modifiable barriers and facilitators through a motivational lens that distinguishes extrinsic from intrinsic motives, mapping intrinsic motives onto autonomy, competence, and relatedness, as proposed by Self-Determination Theory (SDT).

**Methods:**

Searches of MEDLINE, Web of Science, Epistemonikos, and gray literature (inception to June 9, 2025) identified systematic reviews reporting engagement indicators, engagement-outcome associations, or barriers and facilitators among adults with LTCs. Quantitative reviews were appraised using AMSTAR 2 (A Measurement Tool to Assess Systematic Reviews 2), and qualitative and mixed methods reviews were appraised using CASP (Critical Appraisal Skills Programme). A narrative synthesis was undertaken, and modifiable barriers and facilitators were independently mapped by 2 reviewers to extrinsic and intrinsic motivation and, for intrinsic factors, to the SDT constructs of autonomy, competence, and relatedness. Discrepancies were resolved through discussion with the wider research team.

**Results:**

Nineteen reviews (12 quantitative, 5 mixed methods, and 2 qualitative) were included from 4684 records. Fourteen (74%) reviews did not define engagement, and the remaining 5 equated it with “usage” or “adherence,” precluding meta-analysis. Fourteen reviews reported microlevel behavioral indicators, but none captured macrolevel or effective engagement. Eight assessed engagement-outcome links; 7 reported positive associations, and 1 reported no effect. Seven reviews included nonmodifiable factors that influence engagement (eg, ethnicity), while 13 included modifiable factors. SDT mapping revealed that modifiable factors influencing autonomy (eg, personal relevance, flexibility), competence (eg, usability, technical support), and relatedness (eg, clinician endorsement, peer connection) underpin intrinsic engagement, whereas extrinsic barriers include restrictions to access (including cost).

**Conclusions:**

Current evidence on engagement with mHealth apps remains conceptually inconsistent and methodologically fragmented, but motivational patterns are clear: engagement depends largely on intrinsic motives once external conditions are satisfied. Applying SDT provides the first mechanism-oriented explanation of how engagement operates, enabling practical recommendations for evaluating existing apps and designing future mHealth interventions that support autonomy, competence, and relatedness.

## Introduction

Long-term physical health conditions (LTCs) represent a major and growing global challenge for health care systems, driven by their growing prevalence, increasing likelihood of multimorbidity, and rising costs of long-term care [1]. Digital health interventions offer scalable, flexible, and cost-effective potential to enhance LTC self-management and support patient-centered care, particularly in the context of limited health care resources and workforce constraints.

Reflecting this shift, digital health innovation has become a core policy priority. For example, the National Health Service 10 Year Health Plan for England emphasizes the role of digital technologies in empowering patients, improving access to care, and integrating physical and mental health services for people with LTCs [2]. Mobile health (mHealth) apps have garnered particular attention due to their accessibility, portability, and ability to deliver personalized interventions directly to users in real time [3-5]. With the increasing use of smartphones worldwide, mHealth apps can reach diverse populations across geographic and socioeconomic barriers [6]. mHealth apps also support multiple functions relevant to LTC self-management, including symptom monitoring, education, behavior change support, and communication with health care professionals. However, their effectiveness is partly constrained by engagement [7-9].

Engagement is necessary for effectiveness: an mHealth app that is not initiated, is insufficiently used, or is quickly abandoned cannot exert its intended effects [10]. Engagement with digital interventions is multidimensional, comprising behavioral indicators (eg, frequency and depth of use) and subjective indicators, including cognitive (perceived relevance and goal alignment) and affective (eg, interest, enjoyment, or emotional response) components [10,11]. Engagement also operates at multiple levels: the microlevel, capturing moment-to-moment interactions with the intervention (eg, logging in, completing a task), and the macrolevel (users’ internalizing and integrating the intervention’s goals into daily life) [11,12]. Importantly, effective engagement is not about engagement volume. Rather, it concerns the extent and type of engagement necessary to achieve desired outcomes, acknowledging that sustained use may not always be required if the user has internalized the intervention’s purpose [11].

Although engagement has been described in detail using the aforementioned concepts [10-12], systematic reviews show inconsistent definitions and operationalization of engagement, impeding the synthesis of findings and making it difficult to understand if and why app use is sustained and health promoting [13,14]. As a result, there is limited consensus on how engagement should be conceptualized, how it is initiated and sustained over time, and, critically, how much engagement is “enough.” This lack of clarity has contributed to widespread uncertainty about how to design mHealth apps that people will actually use, even when those apps are evidence-based and demonstrably effective under controlled conditions.

A key source of this confusion may be the limited attention paid to the motivational mechanisms underlying engagement. Engagement is often treated as being driven by features such as reminders or clinician oversight [8,11]. While such externally regulated strategies may increase short-term use, they are resource-intensive and difficult to scale, particularly in health care systems seeking to reduce clinician workload and maximize patient self-management [15]. In contrast, engagement is more likely to be sustained when individuals are internally motivated to use an app because they perceive it as personally valuable, relevant, and supportive of their goals [16]. Understanding intrinsic motives is therefore critical for realizing the scalability promise of mHealth app interventions for LTCs.

Self-Determination Theory (SDT) provides a well-established and empirically supported framework for understanding how and why people initiate and maintain behaviors [17]. SDT distinguishes between extrinsic motivation (behavior driven by external demands or rewards) and intrinsic motivation (behavior that is self-endorsed and aligned with personal values). According to SDT, intrinsic motivation is supported when three basic psychological needs—autonomy, competence, and relatedness—are satisfied. This framework is particularly relevant to mHealth apps, where achieving effective engagement often depends on users internalizing the value of app use rather than responding to extrinsic prompts alone [16,18]. Mapping app features and contextual factors onto SDT constructs offers a theoretically grounded, mechanistic understanding of how app design and features (eg, personalization, feedback, social features, and integration with care pathways) may support or undermine engagement.

Although numerous systematic reviews have reported engagement with mHealth apps for LTCs, few have synthesized findings within a coherent motivational framework [19,20]. Existing reviews frequently describe barriers and facilitators of engagement but stop short of explaining why these factors matter or how they relate to intrinsic vs extrinsic motives. This has restricted their ability to inform intervention design, evaluation, and policy, resulting in mixed findings and limited cumulative insight.

To address these gaps, this overview of systematic reviews aimed to (1) synthesize evidence on how engagement with mHealth apps for LTCs is defined, measured, and associated with health outcomes, (2) explore intrinsic and extrinsic motivational processes underlying engagement, and (3) provide practical guidance for developing scalable, user-centered digital health interventions that support effective engagement with minimal reliance on ongoing clinician input. By doing so, this overview integrates existing reviews to move beyond descriptive cataloging of “what” predicts engagement toward a theoretically grounded explanation of “how” and “why” commonly reported factors may influence it.

## Methods

### Ethical Considerations

This study did not involve human participants and therefore did not require ethical approval, in accordance with institutional and national guidelines.

### Design

This overview of systematic reviews adhered to the PRIOR (Preferred Reporting Items for Overviews of Reviews) guidelines (Checklist 1) and was prospectively registered in the PROSPERO (International Prospective Register of Systematic Reviews; CRD42024604784) [21].

### Deviations From Protocol

Three deviations were made to the registered protocol (PROSPERO CRD42024604784) prior to data extraction and synthesis to improve coherence and practical use:

1. Objective 2 in the PROSPERO registration (identify specific mHealth app-based intervention features that are associated with differing levels of engagement) was removed due to insufficient data in the included reviews.
2. A section on nonmodifiable factors influencing engagement (eg, age, sex, and ethnicity) was added to contextualize barriers and facilitators.
3. Modifiable barriers and facilitators of engagement were classified by motivational type (intrinsic or extrinsic), with intrinsically motivating indicators further mapped onto the basic psychological needs of SDT (autonomy, competence, and relatedness). This was done to propose plausible mechanisms through which app features may influence engagement.

### Search Strategy

A comprehensive search was conducted in MEDLINE, Web of Science, Epistemonikos, and gray literature sources (Social Science Research Network, WorldCat, Health Management Information Consortium [Ovid]) from inception to June 9, 2025. Search terms combined keywords and MeSH related to “chronic conditions” and “mobile.” No date or language limits were applied initially. The strategy was developed with domain experts and 2 senior librarians (Multimedia Appendix 1).

### Eligibility Criteria

Systematic reviews were eligible if they

- included adults (≥18 y) with at least one LTC or persistent physical symptoms (eg, chronic pain or fatigue). For the purposes of this review, LTCs were defined as diagnosed physical health conditions that are persistent, require ongoing management, or have long-term consequences for health and functioning
- evaluated mHealth apps delivered via mobile phones or tablets
- reported behavioral engagement indicators, engagement-outcome associations, or barriers or facilitators to engagement

Reviews were excluded if they did not distinguish app-based interventions from other digital tools or focused solely on passive monitoring (eg, wearables). Reviews were therefore excluded when the population was defined solely by hypertension, obesity, isolated symptoms, exposures, risk factors, or health indicators, rather than by a diagnosed long-term physical condition within the scope of the review. This was based on World Health Organization (WHO) and Global Burden of Disease risk-factor frameworks reporting raised blood pressure (hypertension) and high BMI (overweight and obesity) as metabolic risk factors or health indicators rather than eligible long-term physical condition populations in their own right [22,23]. Search terms were intentionally broad to maximize sensitivity and capture related risk-factor literature; however, final inclusion was determined during screening based on how each review defined and reported the condition.

### Study Selection

Search results were imported into EndNote X7 for deduplication [24]. Two reviewers independently screened titles and abstracts using Covidence, an online software tool to manage screening, extraction, and quality assessment for systematic reviews [25]. During screening, inclusion was limited to English, German, Spanish, or Turkish (the languages fluently spoken by the research team) to ensure accurate appraisal. This allowed us to note how many potentially relevant reviews were excluded for language while maintaining screening reliability. Full texts were retrieved for all records deemed potentially relevant by at least 1 reviewer. Ten percent of the full-text reviews were randomly selected for double screening to assess interrater reliability, yielding a Cohen κ of 0.62, which indicates substantial agreement between authors [26]. Discrepancies were resolved through discussion, and the remaining full-text screening was completed by the first author. Reference lists of included reviews were hand searched.

### Data Extraction

Data extraction used a standardized form piloted by the research team. The first author extracted all data, with a random 10% independently checked by a second reviewer (GHB), yielding full agreement. The remaining data extraction was completed by the first author.

### Quality Appraisal

Quantitative systematic reviews were appraised using the AMSTAR 2 (A Measurement Tool to Assess Systematic Reviews 2) tool and categorized as high, moderate, low, or critically low [27]. Qualitative and mixed methods reviews were appraised using the CASP (Critical Appraisal Skills Programme) qualitative checklist [28]. CASP does not provide a numeric scoring system and instead uses descriptive summaries with justifications. We considered the breadth of “yes” responses, with particular emphasis on core methodological areas, including clarity of aims, appropriateness of qualitative methodology and research design, adequacy of data collection, rigor of data analysis, clarity of findings, and the stated value of the research. We did not apply a prespecified numerical cutoff for most CASP items. Instead, reviews were judged as higher quality when “yes” responses were consistent across these core domains and when any limitations were minor or related to less applicable domains for systematic reviews, such as recruitment strategy. A random 10% were double checked, with full agreement. We considered alternative tools for quality appraisal, including the Joanna Briggs Institute (JBI) checklist for umbrella reviews. However, because our overview included qualitative and mixed methods reviews, we judged CASP to provide more explicit prompts for assessing the interpretive and methodological features of qualitative synthesis. CASP was therefore used as a structured framework to appraise the qualitative synthesis components of the included reviews.

### Data Synthesis

A narrative synthesis was undertaken. Reviews were grouped according to the engagement-related data they reported:

1. Behavioral indicators of engagement (amount, duration, depth, and frequency) [10,11]
2. Associations between engagement and health outcomes
3. Nonmodifiable barriers and facilitators of engagement (eg, age and ethnicity)
4. Modifiable barriers and facilitators of engagement (eg, digital literacy and app design)

Where definitions were absent, engagement-related terms (eg, usage, adherence, and compliance) were interpreted using the context from primary studies. For consistency in characterizing core mHealth app functionalities, we applied the WHO classification of digital health interventions [29].

Synthesis of modifiable barriers and facilitators offered insight into users’ cognitive and affective responses to mHealth apps, reflecting how perceptions (eg, relevance and trust) and emotions (eg, interest and burden) influenced decisions to engage or disengage. This enabled exploration of subjective influences on engagement.

Modifiable barriers and facilitators of engagement were categorized as intrinsic or extrinsic motives. The intrinsic motives were then systematically mapped onto the 3 basic psychological needs proposed by SDT—autonomy, competence, and relatedness—using established definitions of SDT psychological needs [17].

- Autonomy: experiences of volition, choice, and personal relevance
- Competence: perceptions of efficacy, capability, and mastery
- Relatedness: feelings of connection, being cared for, and a sense of belonging with others

Mapping was completed independently by 2 reviewers (JB and MMI). Decisions about which barriers and facilitators aligned with each SDT need were informed by existing theoretical and empirical literature [30,31] and refined through iterative discussions within the research team.

The frequency with which themes were mapped to each psychological need was descriptively recorded across reviews and their included primary studies to enhance the transparency of the synthesis rather than to infer effect size or relative importance.

Although the Capability, Opportunity, Motivation-Behavior (COM-B) model was initially considered as an organizing framework [32], preliminary synthesis showed that most reviews described engagement processes in terms of the quality of motivation rather than broader behavioral determinants, such as physical capability or environmental opportunity. Common descriptors included perceived personal relevance and feelings of being supported, which align more closely with SDT’s distinction between intrinsic and extrinsic motivation. Given that sustaining engagement with mHealth apps typically relies on intrinsic, self-endorsed motivation rather than externally regulated behavior [17], SDT provided a more coherent and mechanistic framework for interpreting engagement processes.

Primary study overlap across the included systematic reviews was assessed using a citation matrix. Rows represented unique primary studies, and columns represented included systematic reviews. Overlap was quantified descriptively by identifying primary studies that appeared in more than 1 review and by calculating the corrected covered area (CCA) [33]. As this overview of systematic reviews synthesized data at the systematic review level, primary study overlap was used to inform transparency and interpretation only; no primary study-level outcome data were extracted, reanalyzed, or pooled.

## Results

### Search Results

**Figure 1.**
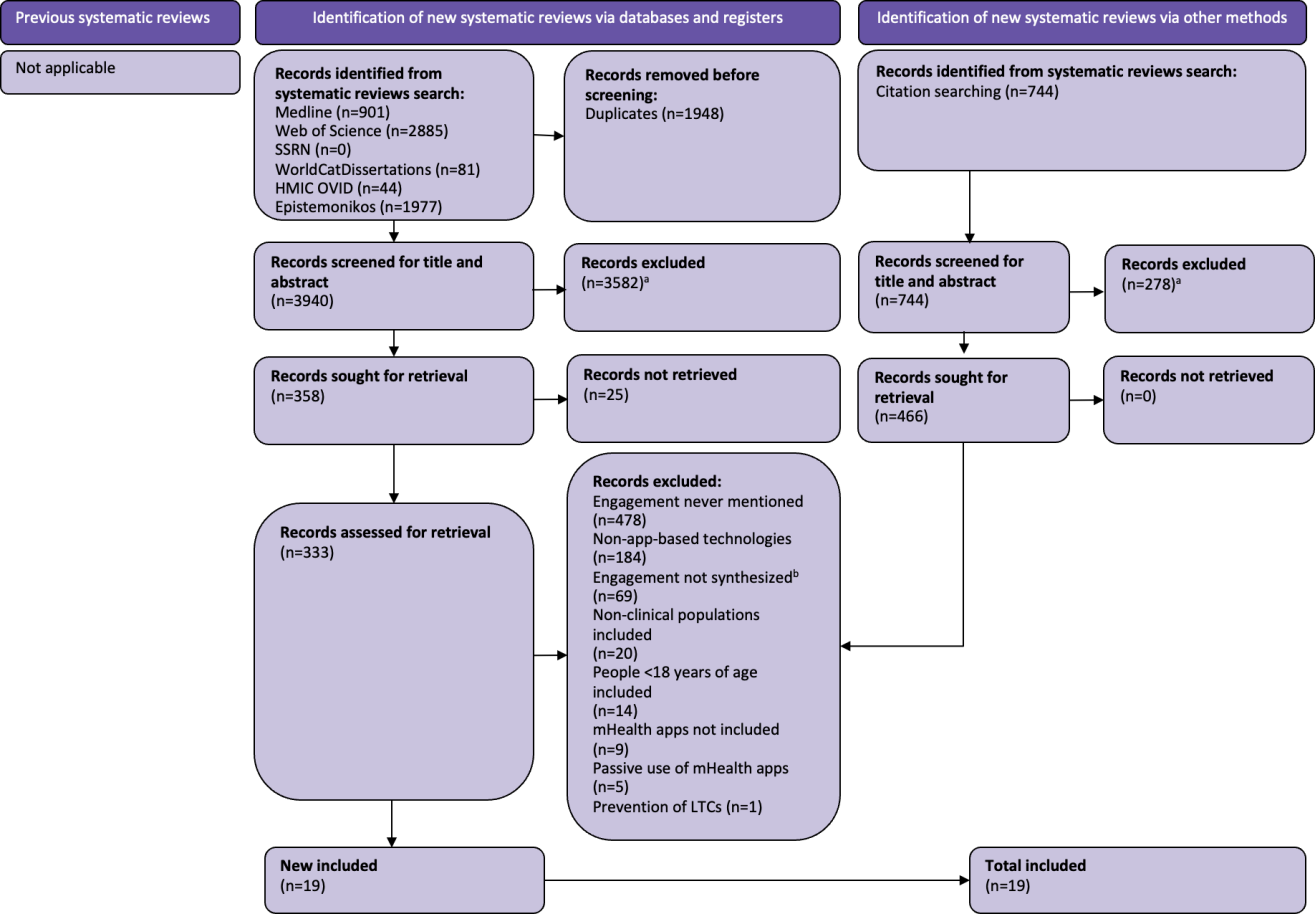
PRIOR (Preferred Reporting Items for Overviews of Reviews) flow diagram of the study selection process. ^a^Reviews were of long-term risk factors (obesity, hypertension, etc) and/or transitory conditions (eg, gestational diabetes mellitus) (n=197) and/or searched app stores only (n=45). ^b^The primary articles included measured engagement, but this was not synthesized in the review. SSRN: Social Science Research Network.

The search identified 5888 records from databases and registers and 744 through citation searching. After removing duplicates, 4684 records including 3940 (84.1%) from databases and registers and 744 (15.9%) from other methods. Overall, 799 full-text articles were assessed for eligibility by 2 independent reviewers, of which 333 (41.7%) were identified through databases and registers and 466 (58.3%) through other methods. The primary reason for exclusion (n=478, 59.9%) was the absence of any mention or reporting of engagement-related data. Ultimately, 19 systematic reviews were included in this overview of systematic reviews [34-52]. A PRIOR (Preferred Reporting Items for Overviews of Reviews) flow diagram is presented in Figure 1.

### Characteristics of Included Reviews

The 19 systematic reviews comprised 12 (63%) quantitative, 5 (26%) mixed methods, and 2 (11%) qualitative syntheses published between 2015 and 2025. Of the 17 reviews with quantitative data, 3 (18%) conducted a meta-analysis. Of the 7 reviews with qualitative data, 1 (14%) undertook a formal meta-synthesis, while the remainder used thematic or narrative approaches. LTC populations included people living with cancer (n=6, 32%), diabetes (n=4, 21%), and chronic pain (n=2, 11%). Others focused on heart failure (n=1, 5%), stroke (n=1, 5%), chronic kidney disease (n=1, 5%), osteoarthritis (n=1, 5%), Parkinson disease (n=1, 5%), traumatic brain injury (n=1, 5%), and a mixed group of LTCs (n=1, 5%).

The number of primary studies per review ranged from 3 to 30 (total n=262), with sample sizes ranging from 1 to 3606 and mean participant ages typically between 50 and 63 years. Across the 262 primary studies, the three most common study designs were randomized controlled trials (RCTs; n=111, 42.4%), qualitative studies (n=62, 24%), and observational studies (n=33, 13%). Of the 195 primary studies that reported geographic origin, 83 (43%) were conducted in North America, 68 (35%) in Europe, 24 (12%) in Asia, 12 (6%) in Oceania, 4 (2%) in South America, and 4 (2%) in Africa. Table 1 summarizes these characteristics, and Multimedia Appendix 2 provides a more detailed table.

**Table 1.** Characteristics of included systematic reviews.^a^

Author, year	Systematic review characteristics			Primary studies’ characteristics					
	Review type	Quality of reviews	Publication year range	Primary studies	Type of LTC^b^	Study designs for primary studies	Country of studies	Sample size, total (range)	Age (y), mean (range)
Alaslawi et al [36], 2022	Mixed methods	Moderate^c^	2015‐2019	28	Diabetes	Qualitative (n=14), cross-sectional (n=12), cohort (n=1), mixed methods (n=1)	United States (n=10), Canada (n=3), United Kingdom (n=3), Australia (n=2), Saudi Arabia (n=2), Germany (n=2), Peru (n=1), Denmark (n=1), Rwanda (n=1), New Zealand (n=1), Norway (n=1), China (n=1)	Not reported	Not reported
Bezerra Giordan et al [45], 2022	Mixed methods	Moderate-to-high^c^	2012‐2022	28	Heart failure	RCTs^d^ (n=10), quasi-experimental (n=8), qualitative (n=10)	United States (n=15), Canada (n=4)	1397 (5-232)	Mean: 63.4
Campbell and Porter [39], 2015	Quantitative	Critically low^e^	2003‐2013	5	Chronic kidney disease stages 3‐5, including dialysis patients	RCTs (n=2), case studies/case reports (n=3)	United States (n=5)	60 (1 - 44)	Range: 50.3‐70
de Melo Santana et al [40], 2023	Quantitative with meta-analysis	Low^e^	2018‐2022	5	Chronic low back pain	RCTs (n=5)	Germany (n=2), Jordan (n=1), Denmark (n=1), Norway (n=1), India (n=1)	Total: 447	Range: 18‐65
Diez Alvarez et al [47], 2024	Quantitative	Low^e^	2015‐2020	3	Diabetes	Qualitative (n=1), interventional (n=1), observational (n=1)	Not specified	Range: 4‐60	Not reported
Dunham et al [46], 2021	Mixed methods	Moderate^c^	2013‐2020	10	Osteoarthritis with or without chronic pain	RCT (n=1), quasi-experimental (n=1), mixed methods (n=2), qualitative (n=6)	United States, United Kingdom, Australia	Range: 18‐738	Not reported
Frid et al [41], 2024	Quantitative	Moderate^e^	2015‐2023	30	Breast cancer	RCT (n=25), Q-RCT (n=1), quasi-experimental (n=3), observational (n=1)	Not specified	3606 (35-490)	Not reported
He et al [42], 2022	Quantitative with meta-analysis	Critically low^e^	2011‐2020	19	Type 2 diabetes	Multicenter RCT (n=9), others not specified	South Korea, Australia, China, Canada, Mexico, Netherlands, Norway, India, United States, Japan	2585 (54‐247)	52.7 (31.7‐68)
Hernandez Silva et al [43], 2019	Quantitative	Critically low^e^	2008‐2017	7	Cancer (breast cancer [n=4], lung cancer [n=2], colorectal cancer [n=2], prostate cancer [n=1], lymphoma [n=1])	RCTs (n=2), quasi-randomized (n=1), nonrandomized study with control group (n=1), single-arm studies (n=3)	United States (n=3), United Kingdom (n=2), Korea (n=1), Sweden (n=1)	Range: 16‐356	58.5 (50.3‐69)
Horn et al [51], 2025	Quantitative	Low^e^	2005‐2023	11	Breast cancer	RCTs (n=11)	Netherlands (n=6), Japan (n=1), Turkey (n=1), Germany (n=1), United States (n=2)	Total: 2249	Range: 43.9‐56.2
Lee et al [34], 2022	Quantitative	Low^e^	2013‐2020	17	Parkinson disease	Observational (n=12), quasi-experimental (n=2), RCTs (n=3)	United States (n=7), England (n=2), Finland (n=2), Italy (n=2), Netherlands (n=2), United Kingdom (n=2), Australia (n=1), Belgium (n=1), Greece (n=1), Israel (n=1), Scotland (n=1)	Total: 1,246	63.02 (34-84)
MacLean et al [52], 2025	Quantitative	Low^e^	2016‐2022	8	Chronic pain	RCTs (n=6), single-arm trials (n=2)	United States (n=4), Spain (n=1), Germany (n=1), Brazil (n=1), Australia (n=1)	Range: 20‐206	50 (18–85)
Magalhães et al [48], 2021	Quantitative	Critically low^e^	2007‐2019	10	Cancer	Prospective intervention studies (n=6), randomized control studies (n=4) (pilot study [n=1], multicentric clinical trial [n=1])	United Kingdom, Switzerland, South Korea, China	Total: 616	Not reported
O'Neill et al [38], 2022	Qualitative	High^c^	2013‐2019	14	Type 2 diabetes	Mixed methods (n=5), qual element of an RCT (n=3), 1‐1 qualitative (n=5), focus group (n=1)	Norway (n=2), United States (n=5), Germany (n=1), Australia (n=3), United Kingdom (n=2), Canada (n=1)	Total: 248	Range: 24‐80
Patail et al [50], 2025	Qualitative	High^c^	2018‐2023	24	Diabetes	Face-to-face interviews (n=21), telephone interviews (n=2), electronic survey with open-ended questions (n=1)	Asia (n=4), Europe (n=5), United Kingdom (n=2), United States (n=8), Canada (n=1), Central Africa (n=1), multinational (Singapore and Germany) (n=1)	Not reported	Range: 23‐81
Patterson et al [44], 2021	Mixed methods with meta-analysis	Moderate-to-high^c^	2015‐2020	19	Coronary heart disease (n=10), hypertension (n=4), stroke (n=3), heart failure (n=1), peripheral artery disease (n=1)	RCT (n=10), non-RCT (n=3), cohort (n=6)	United States (n=10), Australia (n=1), Spain (n=2), Sweden (n=1), Norway (n=1), Israel (n=1), Scotland (n=1), Multicenter RCT (Spain, Germany, United Kingdom) (n=1), China (n=1), Germany (n=1)	Total: 1,543	59.7 (46.3‐69)
Rintala et al [35], 2023	Quantitative	Low^e^	2021‐2022	11	Stroke survivors (chronic, subacute, and mixed stages)	Controlled clinical trials (n=7; RCT [n=5], non-RCT [n=2]), uncontrolled clinical trials (n=4)	Europe (n=5), United Kingdom (n=1), Spain (n=1), Netherlands (n=1), Israel (n=1), Asia (n=3), South Korea (n=1), Philippines (n=1), North America (n=1), United States (n=1), South America (n=1), Chile (n=1), Africa (n=1), Ghana (n=1)	Total: 264	Median: 59.3 (IQR: 55.3‐61.0)
Vaezipour et al [37], 2019	Mixed methods	High^c^	2015‐2017	4	Moderate-severe traumatic brain injury	Before-after design with preinterview, postinterview, and 2-month postintervention follow-up (n=1), participatory design approach for system development and presystem and postsystem evaluation (n=1), repeated-measures design (n=1), online survey (n=1)	Denmark (n=1), Canada (n=1), United States (n=1), Australia (n=1)	Total: 204	Range: 21‐60
Whitehead and Seaton [49], 2016	Quantitative	Critically low^e^	2008‐2014	9	Diabetes (n=5), chronic lung disease (n=3), cardiovascular disease (n=1)	RCTs (n=9)	Europe (n=3), Oceania (n=2), Asia (n=3), United States (n=1)	Range: 48‐288	Range: 33.8‐72.1

a If the systematic review included data from a population other than adults with long-term conditions, and/or interventions other than mobile health apps, only the data relevant to the overview were extracted.

b LTC: long-term condition.

c Quality of qualitative and mixed methods systematic reviews was rated using the CASP (Critical Appraisal Skills Programme) tool.

d RCT: randomized controlled trial.

e Quality of quantitative systematic reviews was rated using the AMSTAR 2 (A Measurement Tool to Assess Systematic Reviews 2) tool.

### Quality Appraisal

Qualitative and mixed methods reviews were strong overall, with 3 rated “high quality” [37,38,50], 2 rated “moderate-to-high” [44,45], and 2 rated “moderate” [36,46], though reflexivity and analytic transparency were often limited (Table 1).

Quantitative reviews were generally low quality: 6 were rated “critically low” [39,40,42,43,48,49], 5 were rated “low” [34,47,51,52], and 1 was rated “moderate” [41]. None met the criteria for high quality, mainly due to missing protocol registration, limited risk-of-bias assessment, and insufficient reporting of heterogeneity or funding (Table 1). Multimedia Appendices 3 and 4 include the quality appraisal scores for AMSTAR 2 and CASP, respectively.

### Overlap of Primary Studies

Across the 19 included systematic reviews, 262 unique primary studies were identified. Primary study overlap was slight [33]. Most primary studies appeared in only one review (n=243, 92.7%), while 19 studies appeared in more than one review (19/262, 7.3%). Of these overlapping studies, 14 appeared in 2 reviews, 4 appeared in 3 reviews, and 1 appeared in 4 reviews. The total number of primary study occurrences across reviews was 287. The CCA was 0.53%, indicating slight overlap. Given the low level of overlap and because synthesis was conducted at the systematic review level, all 19 reviews were retained in the narrative synthesis.

### Description of mHealth App-Based Interventions

Across the 19 reviews, 98 distinct mHealth apps were reported. The most common app functionality, using the WHO classifications of digital health interventions, was symptom self-monitoring, which was present in 17 (90%) systematic reviews. Targeted delivery of health information, often as educational content tailored to users’ LTCs, was identified in 16 (84%) reviews. Alerts and reminders were reported in 12 (63%) reviews. Active user data capture, primarily for symptom tracking and remote consultations with health care providers, appeared in 8 (42%) reviews. Peer group support features were reported in 5 (26%), while user-to-system feedback mechanisms were present in 2 (11%) reviews. Only 1 (5%) review included apps where users could access their personal health records. Full characteristics are provided in Multimedia Appendix 5.

### Behavioral Engagement Indicators

For clarity, we describe behavioral indicators as microlevel usage metrics (eg, amount, duration, depth, and frequency of use) based on previous literature [10-12].

Of the 19 included systematic reviews, only 5 (26%) explicitly operationalized engagement a priori [36-38,46,50]. Fourteen (74%) reviews reported engagement metrics, but predominantly as microlevel indicators (eg, frequency of logins, duration of use, and number of tasks completed). Terms such as “usage,” “adherence,” and “engagement” were often used interchangeably, with no review operationalizing macrolevel engagement (eg, integration into daily life and alignment with goals). Definitions and thresholds varied widely, precluding meta-analysis. Multimedia Appendix 6 provides a detailed summary of the engagement indicators reported in each review.

### Engagement-Health Outcome Associations

Eight of the 19 (42%) reviews assessed links between engagement and health outcomes. Seven of these reviews (moderate-to-high [n=1], moderate [n=1], low [n=4], and critically low [n=1]) reported positive associations between microlevel app use and outcomes such as improved symptom knowledge, health behavior change, or reduced distress [39,41,45,47,48,51,52]. One meta-analysis (critically low quality) found no significant association [40].

Importantly, no review linked macrolevel engagement with health outcomes or if engagement was sufficient to achieve intended health outcomes (ie, effective engagement) [11]. Qualitative reviews provided insight into subjective engagement indicators, such as users’ sense of motivation or identification with the intervention, but no review integrated these with quantitative outcome data. Behavioral and subjective indicators of engagement were reported descriptively in separate strands in the included reviews, with no further analysis to link them.

### Nonmodifiable Barriers and Facilitators of Engagement

**Figure 2.**
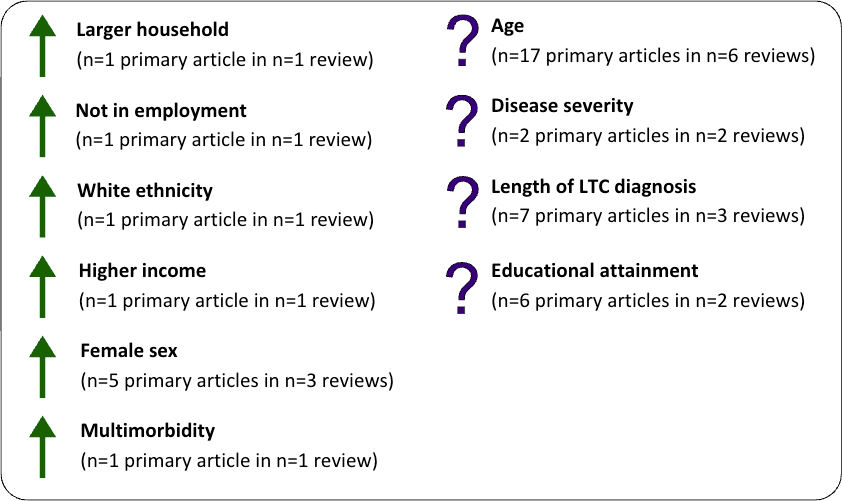
Nonmodifiable barriers and facilitators of engagement with mobile health apps reported in 37% (7/19) of systematic reviews. Upward arrows indicate higher engagement, and question marks indicate mixed findings. LTC: long-term condition.

Seven of the 19 (37%) systematic reviews highlighted nonmodifiable user-specific barriers and facilitators of engagement [36,41,42,45,46,48,52] (Figure 2). A total of 57% (4/7) of reviews were quantitative (moderate [n=1], low [n=1], and critically low quality [n=2]), and 43% (3/7) of reviews were mixed methods (moderate-to-high [n=1] and moderate quality [n=2]).

Findings were mixed, as 2 reviews reported higher engagement among older adults (≥63 years in 1 review; age not specified in the other) [42,46], whereas another reported greater engagement among younger users [36]. Similarly, disease-related characteristics such as duration of diagnosis had mixed results, with both newly diagnosed individuals and those living with a condition for over 5 years demonstrating active engagement across different studies [36,42]. Findings related to disease severity or multimorbidity were also mixed, with one review reporting lower engagement [41] and another reporting higher engagement, potentially due to increased pain burden [40].

### Modifiable Barriers and Facilitators of Engagement

Thirteen of the 19 (68%) reviews reported modifiable barriers and facilitators of engagement with mHealth apps for LTCs [34,36-38,42-46,48-50,52]. Seven reviews were quantitative (moderate [n=1], low [n=2], and critically low quality [n=4]), 4 were mixed methods reviews (moderate-to-high [n=2] and moderate quality [n=2]), and 2 were qualitative reviews (high quality [n=2]).

**Figure 3.**
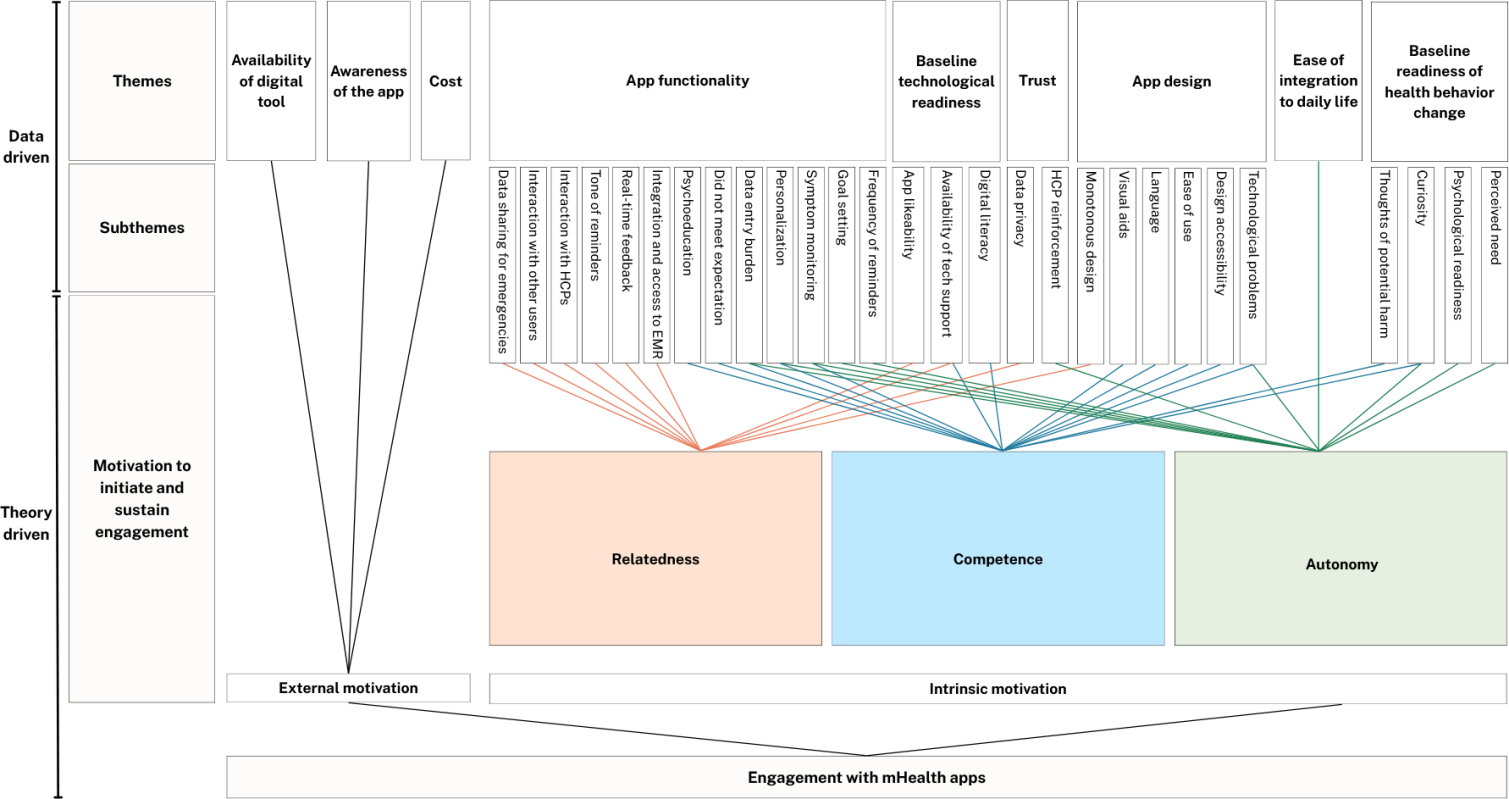
Motivational mechanisms of engagement with mobile health apps. EMR: electronic medical record; HCP: health care professional; mHealth: mobile health.

Reviews were categorized into 9 overarching themes (Figure 3). Six themes mapped onto intrinsic motives: app design, app functionality, trust, time burden, baseline technological readiness, and baseline readiness for health behavior change; and 3 themes mapped onto extrinsic motives: awareness of the app, availability of the digital tool, and cost. The reported extrinsic motives function primarily as structural preconditions for engagement because they enable initial uptake but do not, by themselves, sustain engagement. A full thematic breakdown with illustrative quotations and frequency of reporting is provided in Multimedia Appendix 7.

For intrinsic motivation, barriers and facilitators supporting competence were the most frequently reported, appearing across 63% (12/19) of systematic reviews and 89.3% (234/262) of distinct primary studies. These included app design and functionality features that enabled users to feel capable of using the app effectively, such as ease of use, clear and accessible language, visual aids, real-time feedback, and symptom monitoring. Engagement was undermined when competence was challenged by technical problems, high data entry burden, information overload, unmet expectations, low digital literacy, or concerns that app use could exacerbate psychological distress related to the LTC.

Barriers and facilitators mapping onto autonomy were reported across 58% (11/19) of systematic reviews and 59.5% (156/262) of distinct primary studies. Autonomy-related barriers and facilitators reflected users’ appraisal of relevance, control, and fit with daily life, including readiness for behavior change, perceived need for the app, ability to integrate app use into daily routines, goal-setting functionality, personalization, and privacy considerations.

Factors supporting relatedness were less frequently reported overall, with 53% (10/19) of systematic reviews and 40.5% (106/262) of primary studies, and focused on interpersonal connection and trust. Engagement facilitators included interaction with health care professionals and other users, endorsement by clinicians, secure data sharing with caregivers during emergencies, and integration with electronic medical records. Barriers to relatedness included lack of emotional connection to the app and stigmatizing or overly intrusive reminders.

The mapping of modifiable barriers and facilitators to intrinsic and extrinsic motivation informed the development of design recommendations for future evidence-based mHealth apps for people with LTCs (Table 2). Design implications were generated for all themes identified in the synthesis, irrespective of how frequently they were reported across primary studies. This approach reflects the conceptual aim of this overview of systematic reviews to understand mechanisms of engagement rather than to rank predictors by prevalence. Less frequently reported themes may still represent decisive barriers or facilitators for different user groups, LTCs, or contexts of use, and excluding them would risk overlooking design considerations essential for equitable, scalable, and effective engagement.

**Table 2.** Recommendations for mobile health app–based intervention development that support intrinsic and extrinsic motives.

Motivational category	Key engagement factors identified	Design implications for mHealth^a^ apps
Intrinsic motivation		
Autonomy	Limited perceived need for app use Low psychological readiness for behavior change Time burden and poor fit with daily routines Perceived loss of control due to reminders or data entry demands Privacy concerns	Emphasize personal relevance during onboarding Use nonpressuring language Reduce burden through automation Enable flexible goal setting Provide customizable reminders Ensure transparency over data use Offer tailored features that support user-led engagement
Competence	Technical problems Poor usability or accessibility Information overload Low digital literacy Unmet expectations Anxiety arising from symptom monitoring	Use an accessible and intuitive design Clear and plain-language communication Visual information delivery Emotionally supportive and timely feedback Accurate expectation-setting Ongoing technical support to accommodate varying literacy and skill levels
Relatedness	Limited trust in app content Absence of clinical endorsement Lack of social or emotional connection Stigmatizing or impersonal reminder tone	Embed clinician endorsement Enable optional peer interaction Support secure data sharing with trusted others Integrate with clinical systems Use supportive, nonstigmatizing communication in reminders and pop-up notifications
Extrinsic motivation	Lack of awareness of apps Limited digital access or connectivity Financial barriers	Integrate app into routine care pathways Design for low-connectivity environments (eg, offline functionality, lightweight data use) Ensure core features are freely available and explore reimbursement, subsidy, or health system–supported funding models

a mHealth: mobile health.

## Discussion

### Principal Findings

To our knowledge, this overview of systematic reviews is the first to offer theory-informed, mechanism-oriented guidance for understanding and designing mHealth app–based interventions that support engagement among adults with LTCs. By mapping modifiable barriers and facilitators of engagement onto SDT, we move beyond descriptive lists of factors to explain why these influences matter and how they may support or undermine intrinsic motivation.

This approach aligns closely with the Medical Research Council framework for developing and evaluating complex interventions, which emphasizes the value of theory for specifying mechanisms of action, strengthening intervention design, and enhancing reproducibility. Applying SDT as an overarching motivational theory provides a coherent structure for identifying modifiable targets, linking app design and features to psychological processes that shape engagement, and treating engagement as an intentional, theorized component of an intervention rather than a by-product. The resulting recommendations (Table 2) therefore offer a workable, mechanism-based framework for integrating psychological needs into both the development and evaluation of mHealth interventions.

Across the 19 included systematic reviews, three consistent findings emerged: (1) conceptual ambiguity in engagement definitions, (2) limited and low-quality evidence on engagement-outcome associations, and (3) converging evidence on patterns in modifiable factors influencing engagement.

First, engagement was rarely defined explicitly. Reviews often used terms including “engagement,” “usage,” and “adherence” interchangeably and reported only microlevel behavioral indicators (eg, number of logins or tasks completed). No review operationalized macrolevel engagement (eg, sustained integration into daily life or alignment with goals). Consequently, the current evidence base largely reflects observed usage, limiting insight into whether engagement was clinically relevant to the person using the app.

Second, although 7 of the 8 reviews assessing engagement-outcome links reported positive associations, these findings were drawn from heterogeneous operationalizations of engagement and were often based on critically low-quality quantitative syntheses. Qualitative reviews contributed insight into subjective indicators of engagement, but no review integrated them with behavioral indicators. This methodological divide reflects broader measurement immaturity in engagement research rather than limitations of individual reviews.

Third, when modifiable barriers and facilitators to engagement were mapped to motivational processes, the vast majority were intrinsic—related to autonomy, competence, or relatedness—rather than extrinsic or structural. Most barriers and facilitators reflected whether users experienced the app as personally relevant, manageable, intuitive, trustworthy, and aligned with their own goals and capabilities. Only a small subset of factors was purely extrinsic (eg, awareness, connectivity, and cost), which is still prominent as this is the minimum needed to enable engagement. However, once extrinsic preconditions are met (eg, users can download the app), engagement largely depends on intrinsic motivational processes. Hence, mHealth apps that aim to replace or reduce clinician input must intentionally support these psychological needs.

SDT provided a clear mechanism through which to interpret these patterns, enabling us to identify specific motivational targets and derive design recommendations to guide the development of interventions that are more capable of sustaining engagement without ongoing clinician reinforcement.

### Comparison With Prior Work

Our findings align with longstanding critiques that digital health research often emphasizes “how much” people engage through behavioral indicators while neglecting the quality of engagement and/or the “why” (ie, subjective [cognitive and affective] influences on engagement) [53-55]. Persistent heterogeneity in engagement terminology and measurement constrains the development of cumulative evidence and makes it difficult to establish whether disengagement reflects unmet needs of the user, symptom improvement, or success in achieving the intervention’s goal [10,56-58]. Without distinguishing between these possibilities, establishing cutoffs such as the “minimum effective dose” of engagement also remains elusive.

The positive engagement-outcome associations, such as improved symptom knowledge, health behavior change, or reduced distress, mirror earlier evidence, including Donkin et al [7] findings, but these associations remain difficult to interpret given inconsistent operationalizations and low review quality. Similarly, although persuasive design features (eg, tailoring and feedback) have been shown to improve engagement [8], none of the included reviews in this overview of systematic reviews analyzed user-level engagement or linked design features to specific mechanisms. Collectively, these findings highlight a need for standardized behavioral metrics and improved reporting of how specific app features are linked to engagement.

### Implications

The implications include the evaluation of existing mHealth apps and the development of new mHealth app interventions.

### Evaluation of Existing mHealth Apps

Definitions and measures of behavioral engagement indicators must be standardized. Future research should adopt a validated multidimensional operationalization of engagement that moves beyond single indicators such as logins. We recommend reporting the amount (eg, number of sessions), duration time spent (eg, minutes spent in sessions), depth (eg, number of tasks completed in the same session), and frequency (eg, number of logins) [10,11]. Complementary use of validated instruments such as the Twente Engagement With eHealth Technologies Scale (TWEETS) [59] can capture both behavioral and subjective dimensions of engagement, including interest, attention, and enjoyment. This combined approach is essential for distinguishing mere usage from outcome-relevant engagement.

### Future Intervention Development

As outlined in Table 2, the motivational mechanisms identified, specifically those supporting autonomy, competence, and relatedness, translate into clear, actionable design recommendations. These recommendations connect psychological needs directly to design choices, supporting mechanism-informed intervention development.

Importantly, many autonomy- and competence-related barriers, such as digital exclusion, cost, and low digital literacy, are structural challenges requiring systemic responses. The features summarized in Table 2 include practical solutions such as offering offline or low-bandwidth app versions, SMS text message–based alternatives for users without smartphones, and device-loan schemes to expand access. To support competence among digitally excluded users, guided onboarding, clear tutorials, and accessible interface design (eg, large fonts, high-contrast modes, and multilingual options) are recommended. Moreover, partnerships with community health workers, primary care providers, and advocacy organizations (relatedness-supportive features) can improve trust and contextual relevance for the LTC populations that the mHealth apps aim to serve [60,61]. These measures directly address engagement gaps and help prevent mHealth innovations from exacerbating health inequalities [62-64]. Further work is needed to quantitatively confirm if some of these recommendations do, in fact, make a difference.

### Strengths and Limitations

A key strength of this overview of systematic reviews is its theory-informed synthesis across quantitative, qualitative, and mixed methods reviews, enabling a multidimensional understanding of engagement with mHealth apps. Unlike prior syntheses focused narrowly on behavioral indicators, this review synthesized the barriers and facilitators of engagement within a motivational framework, which offers insight into subjective indicators. Applying SDT provided a coherent framework for mapping barriers and facilitators onto psychological needs and for generating design-oriented recommendations. Methodological rigor was supported through prospective PROSPERO (International Prospective Register of Systematic Reviews) registration and adherence to PRIOR guidelines.

Several limitations should be acknowledged. Review quality varied; quantitative reviews were often low quality, while qualitative and mixed methods reviews were stronger. In addition, the CASP qualitative checklist was developed for the appraisal of primary qualitative studies rather than review-level evidence syntheses. However, we considered it appropriate as a structured framework for assessing the qualitative and mixed methods components of included reviews because it provides methodological depth in domains such as reflexivity, rigor of analysis, credibility of findings, and the value of the research. This approach is also consistent with a previous overview of systematic reviews that used CASP as part of the quality appraisal of systematic reviews of qualitative studies [65]. Key populations, including older adults, people with low digital literacy, and those in rural or underserved settings, were underrepresented, limiting generalizability. Publication bias may also have excluded studies reporting no engagement-outcome associations. Applying SDT introduced some interpretive subjectivity, as most reviews were not theoretically framed; however, mappings were independently cross-checked to enhance consistency. These limitations reduce the certainty of the conclusions but highlight clear priorities for future research.

### Conclusion

mHealth apps are a promising approach to supporting self-management of LTCs, but their effectiveness partly relies on users’ engagement with the intervention and their ability to apply its therapeutic content in daily life. This overview of systematic reviews highlights conceptual and methodological gaps in engagement research and demonstrates the value of applying SDT to understand why engagement occurs and how it can be supported. The SDT-informed design guidance provides a foundation for developing more engaging, equitable, and user-centered mHealth app interventions. Embedding these principles in future evaluation and development can strengthen both user experience and clinical impact and contribute to more consistent, theory-driven progress in digital health.

*The graphical abstract for the article is provided in Multimedia Appendix 8.*

## Supplementary material

10.2196/88382 Multimedia Appendix 1 Search strategy for all databases and gray literature.

10.2196/88382 Multimedia Appendix 2 Characteristics of included systematic reviews.

10.2196/88382 Multimedia Appendix 3 Quality appraisal of quantitative systematic reviews (n=12) using the AMSTAR 2 tool.

10.2196/88382 Multimedia Appendix 4 Quality appraisal of qualitative and mixed methods systematic reviews (n=7) using the CASP tool.

10.2196/88382 Multimedia Appendix 5 Mobile health app–based intervention characteristics (n=19).

10.2196/88382 Multimedia Appendix 6 Descriptive engagement indicators and their impact on health outcomes (n=19).

10.2196/88382 Multimedia Appendix 7 Mapping modifiable barriers and facilitators of engagement to the Self-Determination Theory.

10.2196/88382 Multimedia Appendix 8 Graphical abstract.

10.2196/88382 Checklist 1 PRIOR checklist_Revision Round_20.06.2026.

## Abbreviations

AMSTAR 2: Assessing the Methodological Quality of Systematic Reviews 2
CASP: Critical Appraisal Skills Program
HMIC: Health Management Information Consortium (Ovid)
JBI: Joanna Briggs Institute
LTC: long-term physical health condition
mHealth: mobile health
PRIOR: Preferred Reporting Items for Overviews of Reviews
PRISMA: Preferred Reporting Items for Systematic Reviews and Meta-Analyses
PROSPERO: International Prospective Register of Systematic Reviews
RCT: randomized controlled trial
SDT: Self-Determination Theory
TWEETS: TWente Engagement with Ehealth Technologies Scale
WHO: World Health Organization
