# Supplementary material for: Mechanisms of Engagement With Mobile Health Apps for Adults With Long-Term Conditions: Overview of Systematic Reviews
Source: JMIR Mhealth Uhealth. 2026 Jul 24;14:e88382. doi: 10.2196/88382 (PMC13398183; doi:10.2196/88382)
Supplement: Multimedia Appendix 1 [file mhealth-v14-e88382-s001.docx]

**Supplemental File 1.**Search strategy for all databases and gray literature

**Database:** Ovid MEDLINE
**Interface/platform:** Ovid
**Date searched:** 09 June 2025
**Coverage:** Inception to 09 June 2025
**Search fields:** Multipurpose field .mp. and MeSH subject headings
**Limits/filters applied:** None
**Records retrieved:** 901

The Ovid MEDLINE search combined three concepts:

1. systematic review/meta-analysis terms;
2. digital/mobile/eHealth/mHealth/telehealth intervention terms; and
3. chronic disease, long-term condition, and condition-specific terms.

The search used text-word searching in the Ovid multipurpose field .mp., exploded MeSH subject headings using exp, Boolean operators, truncation, and wildcard symbols.

| 1. | ("systematic*review" or "meta-analysis").mp. [mp=title, book title, abstract, original title, name of substance word, subject heading word, floating sub-heading word, keyword heading word, organism supplementary concept word, protocol supplementary concept word, rare disease supplementary concept word, unique identifier, synonyms, population supplementary concept word, anatomy supplementary concept word] |
| --- | --- |
| 2. | ("chronic" or "long*term condition*" or "persistent disease" or "non*communicable disease" or "NCD" or "incurable disease" or "life?long condition” or “long*standing illness" or "degenerative*" or "LTC*" or "diabetes" or "*thyroid*" or "Addison*" or "metabolic syndrome" or "hyperlipid*" or "obesity" or "hypertension" or "ischemic heart disease" or "angina" or "heart failure" or "cardiomyopathy" or "arrhythmia" or "vascular disease*" or "stroke" or "COPD" or "asthma" or "bronchitis" or "pulmonary fibrosis" or "irritable bowel syndrome" or "IBS" or "Inflammatory bowel disease" or "IBD" or "Crohn*" or "cirrhosis" or "GERD" or "multiple sclerosis" or "epilepsy" or "Amyotrophic lateral sclerosis" or "ALS" or "neurological disorder" or "*arthritis*" or "fibromyalgia" or "cancer*" or "neoplasm*" or "tumor*" or "psoriasis" or "chronic kidney disease" or "CKD" or "renal failure" or "endometriosis" or "cystic fibrosis" or "genetic disorder" or "hereditary disease" or "h?emophilia" or "sickle cell" or "autoimmune" or "*lupus*" or "glaucoma" or "retinopathy" or "coronary artery disease" or "cardiovascular disease" or "liver disease" or "hepatitis" or "pancreatitis" or "Parkinson*" or "connective tissue disease" or "congenital disease" or "immune deficiency" or "macular degeneration" or "Cushing* syndrome" or "lipid disorder*" or "heart disease" or "atherosclerosis" or "emphysema" or "lung disease" or "hepatitis" or "fatty liver disease" or "MS" or "seizure disorder" or "motor neuron? disease" or "Huntington*" or "cerebral palsy" or "osteoporosis" or "bone disease" or "connective tissue disease" or "scoliosis" or "ankylosing spondylitis" or "*dystrophy" or "myositis" or "malignan*" or "carcinoma" or "leukemia" or "lymphoma" or "melanoma” or “eczema" or "dermatitis" or "scleroderma" or "rosacea" or "skin fibrosis" or "cyst" or "congenital heart disease" or "congenital anomaly" or "genetic disorder" or "chromosomal disorder" or "hereditary disease" or "thalassemia" or "immune deficiency" or "autoimmune disease" or "autoimmune disorder" or "vasculitis" or "spinal injur*" or "TBI" or "RSD" or "sarcoidosis" or "*brain injur*" or "brain damage" or "*dialysis*" or "HIV" or "Lou Gehrig* Disease" or "AIDS").mp. [mp=title, book title, abstract, original title, name of substance word, subject heading word, floating sub-heading word, keyword heading word, organism supplementary concept word, protocol supplementary concept word, rare disease supplementary concept word, unique identifier, synonyms, population supplementary concept word, anatomy supplementary concept word] |
| 3. | exp Mobile Applications/ |
| 4. | exp Computers, Handheld/ |
| 5. | exp ecological momentary assessment/ |
| 6. | exp Chronic Disease/ |
| 7. | "mobile app*".mp. [mp=title, book title, abstract, original title, name of substance word, subject heading word, floating sub-heading word, keyword heading word, organism supplementary concept word, protocol supplementary concept word, rare disease supplementary concept word, unique identifier, synonyms, population supplementary concept word, anatomy supplementary concept word] |
| 8. | "*phone app*".mp. [mp=title, book title, abstract, original title, name of substance word, subject heading word, floating sub-heading word, keyword heading word, organism supplementary concept word, protocol supplementary concept word, rare disease supplementary concept word, unique identifier, synonyms, population supplementary concept word, anatomy supplementary concept word] |
| 9. | "eHealth".mp. [mp=title, book title, abstract, original title, name of substance word, subject heading word, floating sub-heading word, keyword heading word, organism supplementary concept word, protocol supplementary concept word, rare disease supplementary concept word, unique identifier, synonyms, population supplementary concept word, anatomy supplementary concept word] |
| 10. | mHealth.mp. [mp=title, book title, abstract, original title, name of substance word, subject heading word, floating sub-heading word, keyword heading word, organism supplementary concept word, protocol supplementary concept word, rare disease supplementary concept word, unique identifier, synonyms, population supplementary concept word, anatomy supplementary concept word] |
| 11. | "mobile health*".mp. [mp=title, book title, abstract, original title, name of substance word, subject heading word, floating sub-heading word, keyword heading word, organism supplementary concept word, protocol supplementary concept word, rare disease supplementary concept word, unique identifier, synonyms, population supplementary concept word, anatomy supplementary concept word] |
| 12. | "telehealth".mp. [mp=title, book title, abstract, original title, name of substance word, subject heading word, floating sub-heading word, keyword heading word, organism supplementary concept word, protocol supplementary concept word, rare disease supplementary concept word, unique identifier, synonyms, population supplementary concept word, anatomy supplementary concept word] |
| 13. | "e*therap*".mp. [mp=title, book title, abstract, original title, name of substance word, subject heading word, floating sub-heading word, keyword heading word, organism supplementary concept word, protocol supplementary concept word, rare disease supplementary concept word, unique identifier, synonyms, population supplementary concept word, anatomy supplementary concept word] |
| 14. | "ecological momentary assessment*".mp. [mp=title, book title, abstract, original title, name of substance word, subject heading word, floating sub-heading word, keyword heading word, organism supplementary concept word, protocol supplementary concept word, rare disease supplementary concept word, unique identifier, synonyms, population supplementary concept word, anatomy supplementary concept word] |
| 15. | EMA.mp. [mp=title, book title, abstract, original title, name of substance word, subject heading word, floating sub-heading word, keyword heading word, organism supplementary concept word, protocol supplementary concept word, rare disease supplementary concept word, unique identifier, synonyms, population supplementary concept word, anatomy supplementary concept word] |
| 16. | "digital interve*".mp. [mp=title, book title, abstract, original title, name of substance word, subject heading word, floating sub-heading word, keyword heading word, organism supplementary concept word, protocol supplementary concept word, rare disease supplementary concept word, unique identifier, synonyms, population supplementary concept word, anatomy supplementary concept word] |
| 17. | 7 or 8 or 9 or 10 or 11 or 12 or 13 or 14 or 15 or 16 |
| 18. | 3 or 4 or 5 or 17 |
| 19. | 2 or 6 |
| 20. | 1 and 18 and 19 |

**Database:** Epistemonikos
**Interface/platform:** Epistemonikos advanced search
**Date searched:** 09 June 2025
**Coverage:** Inception to date searched
**Search fields:** Title and abstract
**Limits/filters applied:** None
**Records retrieved:** 1,977

The Epistemonikos search combined two concepts:

1. digital/mobile/eHealth/mHealth/telehealth intervention terms; and
2. chronic disease, long-term condition, and condition-specific terms.

The search was run using Boolean operators, phrase searching, truncation, and title/abstract field restrictions.

(title:(("mobile app*" OR "*phone app*" OR "digital*interve*" OR "eHealth" OR "mHealth" OR "mobile health*" OR "telehealth" OR "telemedicine" OR "e*therap*" OR "m*therap*" OR "EMA" OR "ecological momentary assessment*")) OR abstract:(("mobile app*" OR "*phone app*" OR "digital*interve*" OR "eHealth" OR "mHealth" OR "mobile health*" OR "telehealth" OR "telemedicine" OR "e*therap*" OR "m*therap*" OR "EMA" OR "ecological momentary assessment*"))) **AND** (title:(("chronic" OR "long*term condition*" OR "persistent disease" OR "non*communicable disease" OR "NCD" OR "incurable disease" OR "life?long condition" OR "long*standing illness" OR "degenerative*" OR "LTC*" OR "diabetes" OR "*thyroid*" OR "Addison*" OR "metabolic syndrome" OR "hyperlipid*" OR "obesity" OR "hypertension" OR "ischemic heart disease" OR "angina" OR "heart failure" OR "cardiomyopathy" OR "arrhythmia" OR "vascular disease*" OR "stroke" OR "COPD" OR "asthma" OR "bronchitis" OR "pulmonary fibrosis" OR "irritable bowel syndrome" OR "IBS" OR "Inflammatory bowel disease" OR "IBD" OR "Crohn*" OR "cirrhosis" OR "GERD" OR "multiple sclerosis" OR "epilepsy" OR "Amyotrophic lateral sclerosis" OR "ALS" OR "neurological disorder" OR "*arthritis*" OR "fibromyalgia" OR "cancer*" OR "neoplasm*" OR "tumor*" OR "psoriasis" OR "chronic kidney disease" OR "CKD" OR "renal failure" OR "endometriosis" OR "cystic fibrosis" OR "genetic disorder" OR "hereditary disease" OR "h?emophilia" OR "sickle cell" OR "autoimmune" OR "*lupus*" OR "glaucoma" OR "retinopathy" OR "coronary artery disease" OR "cardiovascular disease" OR "liver disease" OR "hepatitis" OR "pancreatitis" OR "Parkinson*" OR "connective tissue disease" OR "congenital disease" OR "immune deficiency" OR "macular degeneration" OR "Cushing* syndrome" OR "lipid disorder*" OR "heart disease" OR "atherosclerosis" OR "emphysema" OR "lung disease" OR "hepatitis" OR "fatty liver disease" OR "MS" OR "seizure disorder" OR "motor neuron? disease" OR "Huntington*" OR "cerebral palsy" OR "osteoporosis" OR "bone disease" OR "connective tissue disease" OR "scoliosis" OR "ankylosing spondylitis" OR "*dystrophy" OR "myositis" OR "malignan*" OR "carcinoma" OR "leukemia" OR "lymphoma" OR "melanoma" OR "eczema" OR "dermatitis" OR "scleroderma" OR "rosacea" OR "skin fibrosis" OR "cyst" OR "congenital heart disease" OR "congenital anomaly" OR "genetic disorder" OR "chromosomal disorder" OR "hereditary disease" OR "thalassemia" OR "immune deficiency" OR "autoimmune disease" OR "autoimmune disorder" OR "vasculitis" OR "spinal injur*" OR "TBI" OR "RSD" OR "sarcoidosis" OR "*brain injur*" OR "brain damage" OR "*dialysis*" OR "HIV" OR "Lou Gehrig* Disease" OR "AIDS")) OR abstract:(("chronic" OR "long*term condition*" OR "persistent disease" OR "non*communicable disease" OR "NCD" OR "incurable disease" OR "life?long condition" OR "long*standing illness" OR "degenerative*" OR "LTC*" OR "diabetes" OR "*thyroid*" OR "Addison*" OR "metabolic syndrome" OR "hyperlipid*" OR "obesity" OR "hypertension" OR "ischemic heart disease" OR "angina" OR "heart failure" OR "cardiomyopathy" OR "arrhythmia" OR "vascular disease*" OR "stroke" OR "COPD" OR "asthma" OR "bronchitis" OR "pulmonary fibrosis" OR "irritable bowel syndrome" OR "IBS" OR "Inflammatory bowel disease" OR "IBD" OR "Crohn*" OR "cirrhosis" OR "GERD" OR "multiple sclerosis" OR "epilepsy" OR "Amyotrophic lateral sclerosis" OR "ALS" OR "neurological disorder" OR "*arthritis*" OR "fibromyalgia" OR "cancer*" OR "neoplasm*" OR "tumor*" OR "psoriasis" OR "chronic kidney disease" OR "CKD" OR "renal failure" OR "endometriosis" OR "cystic fibrosis" OR "genetic disorder" OR "hereditary disease" OR "h?emophilia" OR "sickle cell" OR "autoimmune" OR "*lupus*" OR "glaucoma" OR "retinopathy" OR "coronary artery disease" OR "cardiovascular disease" OR "liver disease" OR "hepatitis" OR "pancreatitis" OR "Parkinson*" OR "connective tissue disease" OR "congenital disease" OR "immune deficiency" OR "macular degeneration" OR "Cushing* syndrome" OR "lipid disorder*" OR "heart disease" OR "atherosclerosis" OR "emphysema" OR "lung disease" OR "hepatitis" OR "fatty liver disease" OR "MS" OR "seizure disorder" OR "motor neuron? disease" OR "Huntington*" OR "cerebral palsy" OR "osteoporosis" OR "bone disease" OR "connective tissue disease" OR "scoliosis" OR "ankylosing spondylitis" OR "*dystrophy" OR "myositis" OR "malignan*" OR "carcinoma" OR "leukemia" OR "lymphoma" OR "melanoma" OR "eczema" OR "dermatitis" OR "scleroderma" OR "rosacea" OR "skin fibrosis" OR "cyst" OR "congenital heart disease" OR "congenital anomaly" OR "genetic disorder" OR "chromosomal disorder" OR "hereditary disease" OR "thalassemia" OR "immune deficiency" OR "autoimmune disease" OR "autoimmune disorder" OR "vasculitis" OR "spinal injur*" OR "TBI" OR "RSD" OR "sarcoidosis" OR "*brain injur*" OR "brain damage" OR "*dialysis*" OR "HIV" OR "Lou Gehrig* Disease" OR "AIDS")))) OR abstract:((title:(("mobile app*" OR "*phone app*" OR "digital*interve*" OR "eHealth" OR "mHealth" OR "mobile health*" OR "telehealth" OR "telemedicine" OR "e*therap*" OR "m*therap*" OR "EMA" OR "ecological momentary assessment*")) OR abstract:(("mobile app*" OR "*phone app*" OR "digital*interve*" OR "eHealth" OR "mHealth" OR "mobile health*" OR "telehealth" OR "telemedicine" OR "e*therap*" OR "m*therap*" OR "EMA" OR "ecological momentary assessment*"))) AND (title:(("chronic" OR "long*term condition*" OR "persistent disease" OR "non*communicable disease" OR "NCD" OR "incurable disease" OR "life?long condition" OR "long*standing illness" OR "degenerative*" OR "LTC*" OR "diabetes" OR "*thyroid*" OR "Addison*" OR "metabolic syndrome" OR "hyperlipid*" OR "obesity" OR "hypertension" OR "ischemic heart disease" OR "angina" OR "heart failure" OR "cardiomyopathy" OR "arrhythmia" OR "vascular disease*" OR "stroke" OR "COPD" OR "asthma" OR "bronchitis" OR "pulmonary fibrosis" OR "irritable bowel syndrome" OR "IBS" OR "Inflammatory bowel disease" OR "IBD" OR "Crohn*" OR "cirrhosis" OR "GERD" OR "multiple sclerosis" OR "epilepsy" OR "Amyotrophic lateral sclerosis" OR "ALS" OR "neurological disorder" OR "*arthritis*" OR "fibromyalgia" OR "cancer*" OR "neoplasm*" OR "tumor*" OR "psoriasis" OR "chronic kidney disease" OR "CKD" OR "renal failure" OR "endometriosis" OR "cystic fibrosis" OR "genetic disorder" OR "hereditary disease" OR "h?emophilia" OR "sickle cell" OR "autoimmune" OR "*lupus*" OR "glaucoma" OR "retinopathy" OR "coronary artery disease" OR "cardiovascular disease" OR "liver disease" OR "hepatitis" OR "pancreatitis" OR "Parkinson*" OR "connective tissue disease" OR "congenital disease" OR "immune deficiency" OR "macular degeneration" OR "Cushing* syndrome" OR "lipid disorder*" OR "heart disease" OR "atherosclerosis" OR "emphysema" OR "lung disease" OR "hepatitis" OR "fatty liver disease" OR "MS" OR "seizure disorder" OR "motor neuron? disease" OR "Huntington*" OR "cerebral palsy" OR "osteoporosis" OR "bone disease" OR "connective tissue disease" OR "scoliosis" OR "ankylosing spondylitis" OR "*dystrophy" OR "myositis" OR "malignan*" OR "carcinoma" OR "leukemia" OR "lymphoma" OR "melanoma" OR "eczema" OR "dermatitis" OR "scleroderma" OR "rosacea" OR "skin fibrosis" OR "cyst" OR "congenital heart disease" OR "congenital anomaly" OR "genetic disorder" OR "chromosomal disorder" OR "hereditary disease" OR "thalassemia" OR "immune deficiency" OR "autoimmune disease" OR "autoimmune disorder" OR "vasculitis" OR "spinal injur*" OR "TBI" OR "RSD" OR "sarcoidosis" OR "*brain injur*" OR "brain damage" OR "*dialysis*" OR "HIV" OR "Lou Gehrig* Disease" OR "AIDS")) OR abstract:(("chronic" OR "long*term condition*" OR "persistent disease" OR "non*communicable disease" OR "NCD" OR "incurable disease" OR "life?long condition" OR "long*standing illness" OR "degenerative*" OR "LTC*" OR "diabetes" OR "*thyroid*" OR "Addison*" OR "metabolic syndrome" OR "hyperlipid*" OR "obesity" OR "hypertension" OR "ischemic heart disease" OR "angina" OR "heart failure" OR "cardiomyopathy" OR "arrhythmia" OR "vascular disease*" OR "stroke" OR "COPD" OR "asthma" OR "bronchitis" OR "pulmonary fibrosis" OR "irritable bowel syndrome" OR "IBS" OR "Inflammatory bowel disease" OR "IBD" OR "Crohn*" OR "cirrhosis" OR "GERD" OR "multiple sclerosis" OR "epilepsy" OR "Amyotrophic lateral sclerosis" OR "ALS" OR "neurological disorder" OR "*arthritis*" OR "fibromyalgia" OR "cancer*" OR "neoplasm*" OR "tumor*" OR "psoriasis" OR "chronic kidney disease" OR "CKD" OR "renal failure" OR "endometriosis" OR "cystic fibrosis" OR "genetic disorder" OR "hereditary disease" OR "h?emophilia" OR "sickle cell" OR "autoimmune" OR "*lupus*" OR "glaucoma" OR "retinopathy" OR "coronary artery disease" OR "cardiovascular disease" OR "liver disease" OR "hepatitis" OR "pancreatitis" OR "Parkinson*" OR "connective tissue disease" OR "congenital disease" OR "immune deficiency" OR "macular degeneration" OR "Cushing* syndrome" OR "lipid disorder*" OR "heart disease" OR "atherosclerosis" OR "emphysema" OR "lung disease" OR "hepatitis" OR "fatty liver disease" OR "MS" OR "seizure disorder" OR "motor neuron? disease" OR "Huntington*" OR "cerebral palsy" OR "osteoporosis" OR "bone disease" OR "connective tissue disease" OR "scoliosis" OR "ankylosing spondylitis" OR "*dystrophy" OR "myositis" OR "malignan*" OR "carcinoma" OR "leukemia" OR "lymphoma" OR "melanoma" OR "eczema" OR "dermatitis" OR "scleroderma" OR "rosacea" OR "skin fibrosis" OR "cyst" OR "congenital heart disease" OR "congenital anomaly" OR "genetic disorder" OR "chromosomal disorder" OR "hereditary disease" OR "thalassemia" OR "immune deficiency" OR "autoimmune disease" OR "autoimmune disorder" OR "vasculitis" OR "spinal injur*" OR "TBI" OR "RSD" OR "sarcoidosis" OR "*brain injur*" OR "brain damage" OR "*dialysis*" OR "HIV" OR "Lou Gehrig* Disease" OR "AIDS")))))

**Database:** Web of Science Core Collection
**Interface/platform:** Web of Science
**Date searched:** 09 June 2026
**Coverage:** Inception to 09 June 2026
**Search fields:** All Fields ALL and Topic TS
**Limits/filters applied:** Document Type: Review Article
**Records retrieved:** 2,885

The Web of Science search combined three concepts:

1. systematic review/meta-analysis terms;
2. digital/mobile/eHealth/mHealth/telehealth intervention terms; and
3. chronic disease, long-term condition, and condition-specific terms.

The search used Boolean operators, phrase searching, truncation, wildcard symbols, Topic searching, All Fields searching, and a document type filter for Review Articles.

| 1 | ALL=(“Systematic* review*” or “meta-analysis”) |
| --- | --- |
| 2 | TS=(("mobile app*" OR "phone*app*" OR "digital*interve*" OR "eHealth" OR "mHealth" OR "mobile health*" OR "telehealth" OR "telemedicine" OR "e*therap*" OR "m*therap*" OR "EMA" OR “ecological momentary assessment”)) |
| 3 | TS=(("chronic" OR "long*term condition*" OR "persistent disease" OR "non*communicable disease" OR "NCD" OR "incurable disease" OR "life?long condition" OR "long*standing illness" OR "degenerative*" OR "LTC*" OR "diabetes" OR "*thyroid*" OR "Addison*" OR "metabolic syndrome" OR "hyperlipid*" OR "obesity" OR "hypertension" OR "ischemic heart disease" OR "angina" OR "heart failure" OR "cardiomyopathy" OR "arrhythmia" OR "vascular disease*" OR "stroke" OR "COPD" OR "asthma" OR "bronchitis" OR "pulmonary fibrosis" OR "irritable bowel syndrome" OR "IBS" OR "inflammatory bowel disease" OR "IBD" OR "Crohn*" OR "cirrhosis" OR "GERD" OR "multiple sclerosis" OR "epilepsy" OR "Amyotrophic lateral sclerosis" OR "ALS" OR "neurological disorder" OR "*arthritis*" OR "fibromyalgia" OR "cancer*" OR "neoplasm*" OR "tumor*" OR "psoriasis" OR "chronic kidney disease" OR "CKD" OR "renal failure" OR "endometriosis" OR "cystic fibrosis" OR "genetic disorder" OR "hereditary disease" OR "h?emophilia" OR "sickle cell" OR "autoimmune" OR "*lupus*" OR "glaucoma" OR "retinopathy" OR "coronary artery disease" OR "cardiovascular disease" OR "liver disease" OR "hepatitis" OR "pancreatitis" OR "Parkinson*" OR "connective tissue disease" OR "congenital disease" OR "immune deficiency" OR "macular degeneration" OR "Cushing* syndrome" OR "lipid disorder*" OR "heart disease" OR "atherosclerosis" OR "emphysema" OR "lung disease" OR "fatty liver disease" OR "MS" OR "seizure disorder" OR "motor neuron? disease" OR "Huntington*" OR "cerebral palsy" OR "osteoporosis" OR "bone disease" OR "connective tissue disease" OR "scoliosis" OR "ankylosing spondylitis" OR "*dystrophy" OR "myositis" OR "malignan*" OR "carcinoma" OR "leukemia" OR "lymphoma" OR "melanoma" OR "eczema" OR "dermatitis" OR "scleroderma" OR "rosacea" OR "skin fibrosis" OR "cyst" OR "congenital heart disease" OR "congenital anomaly" OR "chromosomal disorder" OR "thalassemia" OR "immune deficiency" OR "autoimmune disease" OR "autoimmune disorder" OR "vasculitis" OR "spinal injur*" OR "TBI" OR "RSD" OR "sarcoidosis" OR "*brain injur*" OR "brain damage" OR "*dialysis*" OR "HIV" OR "Lou Gehrig* Disease" OR "AIDS")) |
| 4 | #1 AND #2 AND #3 and Review Article (Document Types) |

**Database:** HMIC
**Interface/platform:** Ovid
**Date searched:** 09 June 2026
**Coverage:** From database inception to date searched
**Search fields:** Multipurpose field .mp. and subject headings where available
**Limits/filters applied:** None
**Records retrieved:** 44

The HMIC search combined three concepts:

1. systematic review/meta-analysis terms;
2. digital/mobile/eHealth/mHealth/telehealth intervention terms; and
3. chronic disease, long-term condition, and condition-specific terms.

| 1. | ("systematic*review" or "meta-analysis").mp. [mp=title, book title, abstract, original title, name of substance word, subject heading word, floating sub-heading word, keyword heading word, organism supplementary concept word, protocol supplementary concept word, rare disease supplementary concept word, unique identifier, synonyms, population supplementary concept word, anatomy supplementary concept word] |
| --- | --- |
| 2. | ("chronic" or "long*term condition*" or "persistent disease" or "non*communicable disease" or "NCD" or "incurable disease" or "life?long condition” or “long*standing illness" or "degenerative*" or "LTC*" or "diabetes" or "*thyroid*" or "Addison*" or "metabolic syndrome" or "hyperlipid*" or "obesity" or "hypertension" or "ischemic heart disease" or "angina" or "heart failure" or "cardiomyopathy" or "arrhythmia" or "vascular disease*" or "stroke" or "COPD" or "asthma" or "bronchitis" or "pulmonary fibrosis" or "irritable bowel syndrome" or "IBS" or "Inflammatory bowel disease" or "IBD" or "Crohn*" or "cirrhosis" or "GERD" or "multiple sclerosis" or "epilepsy" or "Amyotrophic lateral sclerosis" or "ALS" or "neurological disorder" or "*arthritis*" or "fibromyalgia" or "cancer*" or "neoplasm*" or "tumor*" or "psoriasis" or "chronic kidney disease" or "CKD" or "renal failure" or "endometriosis" or "cystic fibrosis" or "genetic disorder" or "hereditary disease" or "h?emophilia" or "sickle cell" or "autoimmune" or "*lupus*" or "glaucoma" or "retinopathy" or "coronary artery disease" or "cardiovascular disease" or "liver disease" or "hepatitis" or "pancreatitis" or "Parkinson*" or "connective tissue disease" or "congenital disease" or "immune deficiency" or "macular degeneration" or "Cushing* syndrome" or "lipid disorder*" or "heart disease" or "atherosclerosis" or "emphysema" or "lung disease" or "hepatitis" or "fatty liver disease" or "MS" or "seizure disorder" or "motor neuron? disease" or "Huntington*" or "cerebral palsy" or "osteoporosis" or "bone disease" or "connective tissue disease" or "scoliosis" or "ankylosing spondylitis" or "*dystrophy" or "myositis" or "malignan*" or "carcinoma" or "leukemia" or "lymphoma" or "melanoma” or “eczema" or "dermatitis" or "scleroderma" or "rosacea" or "skin fibrosis" or "cyst" or "congenital heart disease" or "congenital anomaly" or "genetic disorder" or "chromosomal disorder" or "hereditary disease" or "thalassemia" or "immune deficiency" or "autoimmune disease" or "autoimmune disorder" or "vasculitis" or "spinal injur*" or "TBI" or "RSD" or "sarcoidosis" or "*brain injur*" or "brain damage" or "*dialysis*" or "HIV" or "Lou Gehrig* Disease" or "AIDS").mp. [mp=title, book title, abstract, original title, name of substance word, subject heading word, floating sub-heading word, keyword heading word, organism supplementary concept word, protocol supplementary concept word, rare disease supplementary concept word, unique identifier, synonyms, population supplementary concept word, anatomy supplementary concept word] |
| 3. | exp Mobile Applications/ |
| 4. | exp Computers, Handheld/ |
| 5. | exp ecological momentary assessment/ |
| 6. | exp Chronic Disease/ |
| 7. | "mobile app*".mp. [mp=title, book title, abstract, original title, name of substance word, subject heading word, floating sub-heading word, keyword heading word, organism supplementary concept word, protocol supplementary concept word, rare disease supplementary concept word, unique identifier, synonyms, population supplementary concept word, anatomy supplementary concept word] |
| 8. | "*phone app*".mp. [mp=title, book title, abstract, original title, name of substance word, subject heading word, floating sub-heading word, keyword heading word, organism supplementary concept word, protocol supplementary concept word, rare disease supplementary concept word, unique identifier, synonyms, population supplementary concept word, anatomy supplementary concept word] |
| 9. | "eHealth".mp. [mp=title, book title, abstract, original title, name of substance word, subject heading word, floating sub-heading word, keyword heading word, organism supplementary concept word, protocol supplementary concept word, rare disease supplementary concept word, unique identifier, synonyms, population supplementary concept word, anatomy supplementary concept word] |
| 10. | mHealth.mp. [mp=title, book title, abstract, original title, name of substance word, subject heading word, floating sub-heading word, keyword heading word, organism supplementary concept word, protocol supplementary concept word, rare disease supplementary concept word, unique identifier, synonyms, population supplementary concept word, anatomy supplementary concept word] |
| 11. | "mobile health*".mp. [mp=title, book title, abstract, original title, name of substance word, subject heading word, floating sub-heading word, keyword heading word, organism supplementary concept word, protocol supplementary concept word, rare disease supplementary concept word, unique identifier, synonyms, population supplementary concept word, anatomy supplementary concept word] |
| 12. | "telehealth".mp. [mp=title, book title, abstract, original title, name of substance word, subject heading word, floating sub-heading word, keyword heading word, organism supplementary concept word, protocol supplementary concept word, rare disease supplementary concept word, unique identifier, synonyms, population supplementary concept word, anatomy supplementary concept word] |
| 13. | "e*therap*".mp. [mp=title, book title, abstract, original title, name of substance word, subject heading word, floating sub-heading word, keyword heading word, organism supplementary concept word, protocol supplementary concept word, rare disease supplementary concept word, unique identifier, synonyms, population supplementary concept word, anatomy supplementary concept word] |
| 14. | "ecological momentary assessment*".mp. [mp=title, book title, abstract, original title, name of substance word, subject heading word, floating sub-heading word, keyword heading word, organism supplementary concept word, protocol supplementary concept word, rare disease supplementary concept word, unique identifier, synonyms, population supplementary concept word, anatomy supplementary concept word] |
| 15. | EMA.mp. [mp=title, book title, abstract, original title, name of substance word, subject heading word, floating sub-heading word, keyword heading word, organism supplementary concept word, protocol supplementary concept word, rare disease supplementary concept word, unique identifier, synonyms, population supplementary concept word, anatomy supplementary concept word] |
| 16. | "digital interve*".mp. [mp=title, book title, abstract, original title, name of substance word, subject heading word, floating sub-heading word, keyword heading word, organism supplementary concept word, protocol supplementary concept word, rare disease supplementary concept word, unique identifier, synonyms, population supplementary concept word, anatomy supplementary concept word] |
| 17. | 7 or 8 or 9 or 10 or 11 or 12 or 13 or 14 or 15 or 16 |
| 18. | 3 or 4 or 5 or 17 |
| 19. | 2 or 6 |
| 20. | 1 and 18 and 19 |

**Source:** SSRN
**Interface/platform:** SSRN
**Date searched:** 09 June 2026
**Search fields:** Keyword/basic search, adapted to platform functionality
**Limits/filters applied:** None
**Records retrieved:** 0

The SSRN search used the same core concepts as the main database searches: systematic review/meta-analysis terms, digital/mobile/eHealth/mHealth/telehealth terms, and chronic disease/long-term condition terms. Because SSRN does not support the same structured subject heading and field-search functionality as bibliographic databases, the search was adapted using keyword terms.

| Search | Search strategy |
| --- | --- |
| 1 | ("systematic review" OR "systematic reviews" OR "meta-analysis" OR "meta analyses") AND ("mobile app" OR "mobile apps" OR "phone app" OR "phone apps" OR "digital intervention" OR "digital interventions" OR "eHealth" OR "mHealth" OR "mobile health" OR "telehealth" OR "telemedicine" OR "e-therapy" OR "m-therapy" OR "EMA" OR "ecological momentary assessment") AND ("chronic" OR "long-term condition" OR "long-term conditions" OR "persistent disease" OR "non-communicable disease" OR "NCD" OR "incurable disease" OR "lifelong condition" OR "long-standing illness" OR "degenerative" OR "LTC" OR "diabetes" OR "thyroid" OR "Addison" OR "metabolic syndrome" OR "hyperlipid" OR "obesity" OR "hypertension" OR "ischemic heart disease" OR "angina" OR "heart failure" OR "cardiomyopathy" OR "arrhythmia" OR "vascular disease" OR "stroke" OR "COPD" OR "asthma" OR "bronchitis" OR "pulmonary fibrosis" OR "irritable bowel syndrome" OR "IBS" OR "inflammatory bowel disease" OR "IBD" OR "Crohn" OR "cirrhosis" OR "GERD" OR "multiple sclerosis" OR "epilepsy" OR "amyotrophic lateral sclerosis" OR "ALS" OR "neurological disorder" OR "arthritis" OR "fibromyalgia" OR "cancer" OR "neoplasm" OR "tumor" OR "psoriasis" OR "chronic kidney disease" OR "CKD" OR "renal failure" OR "endometriosis" OR "cystic fibrosis" OR "genetic disorder" OR "hereditary disease" OR "haemophilia" OR "hemophilia" OR "sickle cell" OR "autoimmune" OR "lupus" OR "glaucoma" OR "retinopathy" OR "coronary artery disease" OR "cardiovascular disease" OR "liver disease" OR "hepatitis" OR "pancreatitis" OR "Parkinson" OR "connective tissue disease" OR "congenital disease" OR "immune deficiency" OR "macular degeneration" OR "Cushing syndrome" OR "lipid disorder" OR "heart disease" OR "atherosclerosis" OR "emphysema" OR "lung disease" OR "fatty liver disease" OR "seizure disorder" OR "motor neuron disease" OR "Huntington" OR "cerebral palsy" OR "osteoporosis" OR "bone disease" OR "scoliosis" OR "ankylosing spondylitis" OR "dystrophy" OR "myositis" OR "malignant" OR "carcinoma" OR "leukemia" OR "lymphoma" OR "melanoma" OR "eczema" OR "dermatitis" OR "scleroderma" OR "rosacea" OR "skin fibrosis" OR "cyst" OR "congenital heart disease" OR "congenital anomaly" OR "chromosomal disorder" OR "thalassemia" OR "autoimmune disease" OR "autoimmune disorder" OR "vasculitis" OR "spinal injury" OR "TBI" OR "RSD" OR "sarcoidosis" OR "brain injury" OR "brain damage" OR "dialysis" OR "HIV" OR "Lou Gehrig Disease" OR "AIDS") |

**WorldCat**

**Source:** WorldCat
**Interface/platform:** WorldCat
**Date searched:** 09 June 2026
**Search fields:** Keyword/basic or advanced search, adapted to platform functionality
**Limits/filters applied:** None
**Records retrieved:** 81

The WorldCat search used the same core concepts as the main database searches: systematic review/meta-analysis terms, digital/mobile/eHealth/mHealth/telehealth terms, and chronic disease/long-term condition terms. Because WorldCat does not support the same structured subject heading and field-search functionality as bibliographic databases, the search was adapted using keyword terms.

| Search | Search strategy |
| --- | --- |
| 1 | ("systematic review" OR "systematic reviews" OR "meta-analysis" OR "meta analyses") AND ("mobile app" OR "mobile apps" OR "phone app" OR "phone apps" OR "digital intervention" OR "digital interventions" OR "eHealth" OR "mHealth" OR "mobile health" OR "telehealth" OR "telemedicine" OR "e-therapy" OR "m-therapy" OR "EMA" OR "ecological momentary assessment") AND ("chronic" OR "long-term condition" OR "long-term conditions" OR "persistent disease" OR "non-communicable disease" OR "NCD" OR "incurable disease" OR "lifelong condition" OR "long-standing illness" OR "degenerative" OR "LTC" OR "diabetes" OR "thyroid" OR "Addison" OR "metabolic syndrome" OR "hyperlipid" OR "obesity" OR "hypertension" OR "ischemic heart disease" OR "angina" OR "heart failure" OR "cardiomyopathy" OR "arrhythmia" OR "vascular disease" OR "stroke" OR "COPD" OR "asthma" OR "bronchitis" OR "pulmonary fibrosis" OR "irritable bowel syndrome" OR "IBS" OR "inflammatory bowel disease" OR "IBD" OR "Crohn" OR "cirrhosis" OR "GERD" OR "multiple sclerosis" OR "epilepsy" OR "amyotrophic lateral sclerosis" OR "ALS" OR "neurological disorder" OR "arthritis" OR "fibromyalgia" OR "cancer" OR "neoplasm" OR "tumor" OR "psoriasis" OR "chronic kidney disease" OR "CKD" OR "renal failure" OR "endometriosis" OR "cystic fibrosis" OR "genetic disorder" OR "hereditary disease" OR "haemophilia" OR "hemophilia" OR "sickle cell" OR "autoimmune" OR "lupus" OR "glaucoma" OR "retinopathy" OR "coronary artery disease" OR "cardiovascular disease" OR "liver disease" OR "hepatitis" OR "pancreatitis" OR "Parkinson" OR "connective tissue disease" OR "congenital disease" OR "immune deficiency" OR "macular degeneration" OR "Cushing syndrome" OR "lipid disorder" OR "heart disease" OR "atherosclerosis" OR "emphysema" OR "lung disease" OR "fatty liver disease" OR "seizure disorder" OR "motor neuron disease" OR "Huntington" OR "cerebral palsy" OR "osteoporosis" OR "bone disease" OR "scoliosis" OR "ankylosing spondylitis" OR "dystrophy" OR "myositis" OR "malignant" OR "carcinoma" OR "leukemia" OR "lymphoma" OR "melanoma" OR "eczema" OR "dermatitis" OR "scleroderma" OR "rosacea" OR "skin fibrosis" OR "cyst" OR "congenital heart disease" OR "congenital anomaly" OR "chromosomal disorder" OR "thalassemia" OR "autoimmune disease" OR "autoimmune disorder" OR "vasculitis" OR "spinal injury" OR "TBI" OR "RSD" OR "sarcoidosis" OR "brain injury" OR "brain damage" OR "dialysis" OR "HIV" OR "Lou Gehrig Disease" OR "AIDS") |
