# Supplementary material for: Mechanisms of Engagement With Mobile Health Apps for Adults With Long-Term Conditions: Overview of Systematic Reviews
Source: JMIR Mhealth Uhealth. 2026 Jul 24;14:e88382. doi: 10.2196/88382 (PMC13398183; doi:10.2196/88382)
Supplement: Multimedia Appendix 2 [file mhealth-v14-e88382-s002.docx]

| **Table 1.**Characteristics of included systematic reviews | | | | | | | | | | |
| --- | --- | --- | --- | --- | --- | --- | --- | --- | --- | --- |
| **Systematic review characteristics** | | | | **Primary studies' characteristics** | | | | | | |
| **Author, year** | **Review type** | **Quality of reviews** | **Publication year range** | | **Primary studies** | **Type of LTC** | **Study designs for primary studies** | **Country of studies** | **Sample size** | **Age (years)** |
|  |  |  | |  | (n) |  |  |  | Total (range) | Mean (range) |
| Alaslawi et al., 2022 [28] | Mixed methods | Moderate^a^ | | 2015-2019 | 28 | Diabetes | Qualitative n=14, Cross-sectional n=12, Cohort n=1, Mixed Methods n=1 | USA n=10, Canada n=3, UK n=3, Australia n=2, Saudi Arabia n=2, Germany n=2, Peru n=1, Denmark n=1, Rwanda n=1, New Zealand n=1, Norway n=1, China n=1 | Not reported | Not reported |
| Bezerra Giordan et al., 2022 [37] | Mixed methods | Moderate-to-high^a^ | | 2012-2022 | 28 | Heart failure | RCTs n=10, Quasi Experimental n=8, Qualitative n=10 | USA n=15, Canada n=4 | 1397  (5-232) | Mean: 63.4 |
| Campbell & Porter, 2015 [31] | Quantitative | Critically low**^b^** | | 2003-2013 | 5 | Chronic kidney disease stages 3–5, including dialysis patients. | RCTs n=2, Case studies/case reports n=3 | USA n=5 | 60  (1 - 44) | Range: 50.3-70 |
| de Melo Santana et al., 2023 [32] | Quantitative with meta-analysis | Low**^b^** | | 2018-2022 | 5 | Chronic low back pain | RCTs n=5 | Germany n=2, Jordan n=1, Denmark n=1, Norway n=1, India n=1 | Total: 447 | Range: 18-65 |
| Diez Alvarez et al., 2024 [39] | Quantitative | Low**^b^** | | 2015-2020 | 3 | Diabetes | Qualitative n=1, Interventional n=1, Observational n=1 | Not specified | Range: 4-60 | Not reported |
| Dunham et al., 2021 [38] | Mixed methods | Moderate^a^ | | 2013-2020 | 10 | Osteoarthritis with or without chronic pain | RCT n=1, Quasi-experimental n=1, Mixed methods n=2, Qualitative n=6 | USA, UK, Australia | Range: 18-738 | Not reported |
| Frid et al., 2024 [33] | Quantitative | Moderate**^b^** | | 2015–2023 | 30 | Breast cancer | RCT n=25 Q-RCT n=1 Quasi-experimental n=3, observational n=1 | Not specified | 3,606 (35–490) | Not reported |
| He et al., 2022 [34] | Quantitative with meta-analysis | Critically low**^b^** | | 2011-2020 | 19 | Type 2 diabetes | multi-center RCT n=9, others not specified | South Korea, Australia, China, Canada, Mexico, Netherlands, Norway, India, USA, Japan | 2585 (54–247) | 52.7 (31.7-68) |
| Hernandez Silva, Lawler & Langbecker, 2019 [35] | Quantitative | Critically low**^b^** | | 2008-2017 | 7 | Cancer (breast cancer n=4, lung cancer n=2, colorectal cancer n=2, prostate cancer n=1, lymphoma n=1) | RCTs n=2, quasi-randomized n=1, non-randomized study with control group n=1, single-arm studies n=3 | USA n=3, UK n=2, Korea n=1, Sweden n=1 | Range: 16-356 | 58.5 (50.3-69) |
| Horn et al., 2025 [43] | Quantitative | Low**^b^** | | 2005–2023 | 11 | Breast cancer | RCTs n=11 | Netherlands n=6, Japan n=1, Turkey n=1, Germany n=1, USA n=2 | Total: 2249 | Range: 43.9–56.2 |
| Lee et al., 2022 [26] | Quantitative | Low**^b^** | | 2013-2020 | 17 | Parkinson's disease | Observational n=12, quasi-experimental n=2, RCTs n=3 | United States n=7, England n=2, Finland n=2, Italy n=2, Netherlands n=2, United Kingdom n=2, Australia n=1, Belgium n=1, Greece n=1, Israel n=1, Scotland n=1 | Total: 1,246 | 63.02 (34-84) |
| MacLean et al., 2025 [44] | Quantitative | Low**^b^** | | 2016-2022 | 8 | Chronic pain | RCTs n=6, Single-arm trials n=2 | USA n=4, Spain n=1, Germany n=1, Brazil n=1, Australia n=1 | Range: 20–206 | 50  (18–85) |
| Magalhães et al., 2021 [40] | Quantitative | Critically low**^b^** | | 2007-2019 | 10 | Cancer | Prospective intervention studies n=6, Randomized control studies n=4 (Pilot study n=1, Multicentric clinical trial n=1) | UK, Switzerland, South Korea, China | Total: 616 | Not reported |
| O'Neill et al., 2021 [30] | Qualitative | High^a^ | | 2013-2019 | 14 | Type 2 diabetes | Mixed methods n=5, Qual element of an RCT n=3, 1-1 Qualitative n=5, focus group n=1 | Norway n=2, USA n=5, Germany n=1, Australia n=3, UK n=2, Canada n=1 | Total: 248 | Range: 24-80 |
| Patail et al., 2025 [42] | Qualitative | High^a^ | | 2018-2023 | 24 | Diabetes | Face-to-face interviews n=21, telephone interviews n=2, electronic survey with open-ended questions n=1 | Asia n=4, Europe n=5, UK n=2, USA n=8, Canada n=1, Central Africa n=1, Multinational (Singapore and Germany) n=1 | Not reported | Range: 23–81 |
| Patterson et al., 2021 [36] | Mixed methods with meta-analysis | Moderate-to-high^a^ | | 2015-2020 | 19 | Coronary heart disease n=10, hypertension n=4, stroke n=3, heart failure n=1, peripheral artery disease n=1 | RCT n=10, non-RCT n=3, Cohort n=6 | USA n=10, Australia n=1, Spain n=2, Sweden n=1, Norway n=1, Israel n=1, Scotland n=1, Multi-center RCT (Spain, Germany, UK) n=1, China n=1, Germany n=1 | Total: 1,543 | 59.7 (46.3 – 69) |
| Rintala et al., 2023 [27] | Quantitative | Low**^b^** | | 2021-2022 | 11 | Stroke survivors (chronic, subacute, and mixed stages) | Controlled clinical trials n=7 (RCT n=5, non-RCT n=2), Uncontrolled clinical trials n=4 | Europe n=5, United Kingdom n=1, Spain n=1, Netherlands n=1, Israel n=1, Asia n=3, South Korea n=1, Philippines n=1, North America n=1, USA n=1, South America n=1, Chile n=1, Africa n=1, Ghana n=1 | Total: 264 | Median: 59.3 (IQR: 55.3 -61.0) |
| Vaezipour et al., 2019 [29] | Mixed methods | High^a^ | | 2015-2017 | 4 | Moderate-severe traumatic brain injury | Before-after design with pre-, post interview, and 2-month post-intervention follow-up n=1, Participatory design approach for system development and pre and post system evaluation n=1, Repeated-measures design n=1, online survey n=1 | Denmark n=1, Canada n=1, USA n=1, Australia n=1 | Total: 204 | Range: 21-60 |
| Whitehead & Seaton, 2016 [41] | Quantitative | Critically low**^b^** | | 2008–2014 | 9 | Diabetes n=5, chronic lung disease n=3, cardiovascular disease n=1 | RCTs n=9 | Europe n=3, Oceania n=2, Asia n=3, USA n=1 | Range: 48-288 | Range: 33.8-72.1 |
| ^a^Quality of qualitative and mixed methods systematic reviews were rated using the CASP tool.  ^b^Quality of quantitative systematic reviews were rated using the AMSTAR2 tool.  If the systematic review included data from population other than adults with LTCs, and/or interventions other than mHealth apps, only the data relevant to the overview were extracted. | | | | | | | | | | |
