# Supplementary material for: Mechanisms of Engagement With Mobile Health Apps for Adults With Long-Term Conditions: Overview of Systematic Reviews
Source: JMIR Mhealth Uhealth. 2026 Jul 24;14:e88382. doi: 10.2196/88382 (PMC13398183; doi:10.2196/88382)
Supplement: Multimedia Appendix 3 [file mhealth-v14-e88382-s003.docx]

| **Supplemental File 2.** Quality appraisal of quantitative systematic reviews (n=12/19) using the AMSTAR2 tool | | | | | | | | | | | | |
| --- | --- | --- | --- | --- | --- | --- | --- | --- | --- | --- | --- | --- |
| Item | Campbell & Porter, 2015 | de Melo Santana et al., 2023 | Diez Alvarez et al., 2024 | Frid et al., 2024 | He et al., 2022 | Hernandez Silva, Lawler & Langbecker, 2019 | Horn et al., 2025 | Lee et al., 2022 | MacLean et al., 2025 | Magalhães et al., 2021 | Rintala et al., 2023 | Whitehead & Seaton, 2016 |
| **1. Did the research questions and inclusion criteria for the review include the components of PICO?** | | | | | | | | | | | | |
| Population | Y | Y | Y | Y | Y | Y | Y | Y | Y | Y | Y | Y |
| Intervention | Y | Y | Y | Y | Y | Y | Y | Y | Y | Y | Y | Y |
| Comparator group | NA | Y | Y | NA | Y | NA | NA | NA | NA | NA | NA | NA |
| Outcome | Y | Y | Y | Y | Y | Y | Y | Y | Y | Y | Y | Y |
| (Optional) Timeframe for follow-up |  |  |  |  |  |  |  |  |  |  |  |  |
| *Code result: 0 = N, 1 = Y, NA* | 1 | 1 | 1 | 1 | 1 | 1 | 1 | 1 | 1 | 1 | 1 | 1 |
| **2. Did the report of the review contain an explicit statement that the review methods were established prior to the conduct of the review and did the report justify any significant deviations from the protocol?** | | | | | | | | | | | | |
| *For partial Y: The authors state that they had a written protocol or guide that included ALL the following:* | | | | | | | | | | | | |
| The authors state that they had a written protocol or guide, including (below) |  |  |  |  | N |  |  |  |  | N | N | N |
| review question(s) | Y | Y | Y |  |  | Y | Y | Y | Y |  |  |  |
| a search strategy | Y | Y | Y |  |  | Y | Y | Y | Y |  |  |  |
| inclusion/exclusion criteria | Y | Y | Y |  |  | Y | Y | Y | Y |  |  |  |
| a risk of bias assessment | Y | Y | Y |  |  | Y | Y | Y | Y |  |  |  |
| *For Y, ALSO ALL* |  |  |  |  |  |  |  |  |  |  |  |  |
| the protocol should be registered | Y | Y | Y | Y | N | Y | Y | Y | N | N |  |  |
| a meta-analysis/synthesis plan, if appropriate, and | NA | Y | NA | NA |  | NA | Y | NA | NA |  |  |  |
| a plan for investigating causes of heterogeneity | NA | Y | Y | Y |  | NA | Y | NA | N |  |  |  |
| justification for any deviations from the protocol | NA | Y | NA | NA |  | Y | Y | NA | N |  |  |  |
| *Code result: 0 = N, 1 = Y, NA* | 1 | 1 | 1 | 1 | 0 | 1 | 1 | 1 | Partial | 0 | 0 | 0 |
| **3. Did the review authors explain their selection of the study designs for inclusion in the review?**  *For Y, EITHER* | | | | | | | | | | | | |
| Explanation for including only RCTs | Y | Y |  |  | Y |  | Y |  |  |  |  | Y |
| **OR** Explanation for including only NRSI |  |  | Y |  |  |  |  |  |  |  |  |  |
| **OR** Explanation for including both RCTs and NRSI |  |  |  | Y |  | Y |  | Y | Y | Y | Y |  |
| *Code result: 0 = N, 1 = Y, NA* | 1 | 1 | 1 | 1 | 1 | 1 | 1 | 1 | 1 | 1 | 1 | 1 |
| **4. Did the review authors use a comprehensive literature search strategy?** | | | | | | | | | | | | |
| *For Partial Y, ALL* | | | | | | | | | | | | |
| searched at least 2 databases (relevant to research question) | Y | Y | Y | Y | Y | Y | Y | Y | Y | Y | Y | Y |
| provided keyword and/or search strategy | Y | Y | Y | Y | Y | Y | Y | Y | Y | Y | Y | Y |
| justified publication restrictions (e.g. language) | Y | Y | Y | Y | Y | Y | Y | Y | Y | Y | Y | Y |
| *For Y, ALSO ALL* |  |  |  |  |  |  |  |  |  |  |  |  |
| searched the reference lists / bibliographies of included studies | Y | Y | Y | Y | Y | Y | Y | Y | Y | N | Y | Y |
| searched trial/study registries | N | N | N | NA | NA | NA | N | NA | N | N | N | Y |
| included/consulted content experts in the field | N | Y | N | N | N | N | N | Y | N | N | N | N |
| where relevant, searched for gray literature | N | Y | Y | N | N | N | N | N | N | N | N | Y |
| conducted search within 24 months of completion of the review | Y | Y | Y | Y | N | Y | Y | Y | Y | Y | Y | Y |
| *Code result: 0 = N, 1 = Y, NA* | Partial | Partial | Partial | Partial | Partial | Partial | Partial | Partial | Partial | Partial | Partial | Partial |
| **5. Did the review authors perform study selection in duplicate?** | | | | | | | | | | | | |
| *For Y, EITHER* | | | | | | | | | | | | |
| at least two reviewers independently agreed on selection of eligible studies and achieved consensus on which studies to include | Y | Y | Y | Y | Y | N | Y | Y | Y | Y | Y | Y |
| **OR** two reviewers selected a sample of eligible studies and achieved good agreement (at least 80 percent), with the remainder selected by one reviewer. |  |  |  |  |  | N |  |  |  |  |  |  |
| *Code result: 0 = N, 1 = Y, NA* | 1 | 1 | 1 | 1 | 1 | 0 | 1 | 1 | 1 | 1 | 1 | 1 |
| **6. Did the review authors perform data extraction in duplicate?** | | | | | | | | | | | | |
| *For Y, EITHER* | | | | | | | | | | | | |
| at least two reviewers achieved consensus on which data to extract from included studies | Y | Y | N | Y | Y | N | Y | Y | Y | Y | N | N |
| **OR** two reviewers extracted data from a sample of eligible studies and achieved good agreement (at least 80 percent), with the remainder extracted by one reviewer. |  |  |  |  |  | N |  |  |  |  |  |  |
| *Code result: 0 = N, 1 = Y, NA* | 1 | 1 | 1 | 1 | 1 | 0 | 1 | 1 | 1 | 1 | 0 | 0 |
| **7. Did the review authors provide a list of excluded studies and justify the exclusions?** | | | | | | | | | | | | |
| *For Partial Y, ALL* | | | | | | | | | | | | |
| provided a list of all potentially relevant studies that were read in full-text form but excluded from the review | Y | Y | Y | Y | Y | Y | Y | Y | Y | Y | Y | Y |
| *For Y, ALSO ALL* |  |  |  |  |  |  |  |  |  |  |  |  |
| Justified the exclusion from the review of each potentially relevant study | Y | Y | Y | N | Y | Y | Y | Y | Y | Y | Y | Y |
| *Code result: 0 = N, 1 = Y, NA* | 1 | 1 | 1 | Partial | 1 | 1 | 1 | 1 | 1 | 1 | 1 | 1 |
| **8. Did the review authors describe the included studies in adequate detail?** | | | | | | | | | | | | |
| *For Partial Y, ALL* | | | | | | | | | | | | |
| described populations | Y | Y | Y | Y | Y | Y | Y | Y | Y | Y | Y | Y |
| described interventions | Y | Y | Y | Y | Y | Y | Y | Y | Y | Y | Y | Y |
| described comparators | NA | Y | NA | NA | Y | NA | NA | NA | NA | NA | NA | Y |
| described outcomes | Y | Y | Y | Y | Y | Y | Y | Y | Y | Y | Y | Y |
| described research designs | Y | Y | Y | Y | Y | Y | Y | Y | Y | Y | Y | Y |
| *For Y, ALSO ALL* | | | | | | | | | | | | |
| described population in detail | Y | Y | Y | Y | Y | Y | Y | Y | Y | Y | Y | Y |
| described intervention in detail (including doses where relevant) | Y | Y | Y | Y | Y | Y | Y | Y | Y | Y | Y | Y |
| described comparator in detail (including doses where relevant) | NA | Y | NA | NA | Y | NA | NA | NA | NA | NA | NA | Y |
| described study’s setting | Y | Y | Y | N | Y | Y | Y | Y | Y | Y | Y | Y |
| timeframe for follow-up | NA | NA | NA | NA | Y | Y | NA | NA | Y | Y | NA | Y |
| *Code result: 0 = N, 1 = Y, NA* | 1 | 1 | 1 | Partial | 1 | 1 | 1 | 1 | 1 | 1 | 1 | 1 |
| **9. Did the review authors use a satisfactory technique for assessing the risk of bias (RoB) in individual studies that were included in the review?** | | | | | | | | | | | | |
| *For RCTs included in reviews they assess:* | | | | | | | | | | | | |
| *For Partial Y, ALL* | | | | | | | | | | | | |
| unconcealed allocation, and | N | Y | NA | Y | Y | N | Y | Y | Y | Y | Y | Y |
| lack of blinding of patients and assessors when assessing outcomes (unnecessary for objective outcomes such as all-cause mortality) | N | N | NA | Y | N | Y | Y | Y | Y | Y | Y | Y |
| *For Y, ALSO ALL* | | | | | | | | | | | | |
| allocation sequence that was not truly random, and | N | Y | NA | Y | Y | N | Y | Y | Y | N | N | Y |
| selectiveness of the reported result from among multiple measurements or analyses of a specified outcome | N | N | NA | Y | N | N | Y | Y | Y | N | N | Y |
| *For NRSI included in reviews:* |  |  |  |  |  |  |  |  |  |  |  |  |
| *For Partial Y, ALL* |  |  |  |  |  |  |  |  |  |  |  |  |
| from confounding, and | NA | NA | Y | Y | NA | N |  | Y | Y | Y | Y | NA |
| from selection bias | NA | NA | Y | Y | NA | Y |  | Y | Y | Y | Y | NA |
| *For Y, ALSO ALL* |  |  |  |  |  |  |  |  |  |  |  |  |
| methods used to ascertain exposures and outcomes, and | NA | NA | N | Y | NA | N |  | Y | Y | N | N | NA |
| selection of the reported result from among multiple measurements or analyses of a specified outcome | NA | NA | N | Y | NA | Y |  | Y | Y | N | N | NA |
| *Code result: 0 = N, 1 = Y, NA* | 0 | 0 | Partial | 1 | 0 | 0 | 1 | 1 | 1 | Partial | Partial | 1 |
| **10. Did the review authors report on the sources of funding for the studies included in the review?** | | | | | | | | | | | | |
| *For Y, ALL* | | | | | | | | | | | | |
| Must have reported on the sources of funding for individual studies included in the review. Note: Reporting that the reviewers looked for this information but it was Not reported by study authors also qualifies | N | Y | N | N | N | Y | N | N | N | N | N | N |
| *Code result: 0 = N, 1 = Y, NA* | 0 | 1 | 0 | 0 | 0 | 1 | 0 | 0 | 0 | 0 | 0 | 0 |
| **11. If meta-analysis was performed did the review authors use appropriate methods for statistical combination of results?** | | | | | | | | | | | | |
| *For RCTs included in reviews:* | | | | | | | | | | | | |
| *For Y, ALL* | | | | | | | | | | | | |
| The authors justified combining the data in a meta-analysis | NA | Y | NA | NA | Y | NA | NA | NA | NA | NA | NA | NA |
| AND they used an appropriate weighted technique to combine study results and adjusted for heterogeneity if present. | NA | Y | NA | NA | Y | NA | NA | NA | NA | NA | NA | NA |
| AND investigated the causes of any heterogeneity | NA | Y | NA | NA | Y | NA | NA | NA | NA | NA | NA | NA |
| *For NRSI included in reviews:* |  |  |  |  |  |  |  |  |  |  |  |  |
| *For Y, ALL* |  |  |  |  |  |  |  |  |  |  |  |  |
| The authors justified combining the data in a meta-analysis | NA | NA | NA | NA | Y | NA | NA | NA | NA | NA | NA | NA |
| AND they used an appropriate weighted technique to combine study results, adjusting for heterogeneity if present | NA | NA | NA | NA | Y | NA | NA | NA | NA | NA | NA | NA |
| AND they statistically combined effect estimates from NRSI that were adjusted for confounding, rather than combining raw data, or justified combining raw data when adjusted effect estimates were not available | NA | NA | NA | NA | Y | NA | NA | NA | NA | NA | NA | NA |
| AND they reported separate summary estimates for RCTs and NRSI separately when both were included in the review | NA | NA | NA | NA | Y | NA | NA | NA | NA | NA | NA | NA |
| *Code result: 0 = N, 1 = Y, NA* | NA | 1 | NA | NA | 1 | NA | NA | NA | NA | NA | NA | NA |
| **12. If meta-analysis was performed, did the review authors assess the potential impact of RoB in individual studies on the results of the meta-analysis or other evidence synthesis?** | | | | | | | | | | | | |
| *For Y, EITHER* | | | | | | | | | | | | |
| included only low risk of bias RCTs | NA | N | NA | NA | Y | NA | NA | NA | NA | NA | NA | NA |
| **OR**, if the pooled estimate was based on RCTs and/or NRSI at variable RoB, the authors performed analyses to investigate possible impact of RoB on summary estimates of effect. | NA | N | NA | NA | N | NA | NA | NA | NA | NA | NA | NA |
| *Code result: 0 = N, 1 = Y, NA* | NA | 0 | NA | NA | 1 | NA | NA | NA | NA | NA | NA | NA |
| **13. Did the review authors account for RoB in individual studies when interpreting/ discussing the results of the review?** | | | | | | | | | | | | |
| *For Y, EITHER* | | | | | | | | | | | | |
| included only low risk of bias RCTs | N |  | N |  | N | N | N | N | N | N |  | N |
| **OR**, if RCTs with moderate or high RoB, or NRSI were included the review provided a discussion of the likely impact of RoB on the results | N | Y | N | Y | N | N | N | N | N | N | Y | N |
| *Code result: 0 = N, 1 = Y, NA* | 0 | 1 | 0 | 1 | 0 | 0 | 0 | NA | 0 | 0 | 1 | 0 |
| **14. Did the review authors provide a satisfactory explanation for, and discussion of, any heterogeneity observed in the results of the review?** | | | | | | | | | | | | |
| *For Y, EITHER* | | | | | | | | | | | | |
| There was N significant heterogeneity in the results | N |  | N |  |  | N | N | N | N | N |  |  |
| **OR** if heterogeneity was present the authors performed an investigation of sources of any heterogeneity in the results and discussed the impact of this on the results of the review | N | Y | N | Y | Y | N | N | N | N | N | N | Y |
| *Code result: 0 = N, 1 = Y, NA* | 0 | 1 | 0 | 1 | 1 | 1 | 0 | NA | 0 | 0 | 0 | 1 |
| **15. If they performed quantitative synthesis did the review authors carry out an adequate investigation of publication bias (small study bias) and discuss its likely impact on the results of the review?** | | | | | | | | | | | | |
| *For Y, ALL* | | | | | | | | | | | | |
| performed graphical or statistical tests for publication bias and discussed the likelihood and magnitude of impact of publication bias | NA | Y | NA | NA | N | NA | NA | NA | NA | NA | NA | NA |
| *Code result: 0 = N, 1 = Y, NA* | NA | 1 | NA | NA | 0 | NA | NA | NA | NA | NA | NA | NA |
| **16. Did the review authors report any potential sources of conflict of interest, including any funding they received for conducting the review?** | | | | | | | | | | | | |
| *For Y, EITHER* | | | | | | | | | | | | |
| The authors reported N competing interests | Y | Y | Y |  | Y | Y | Y | Y | Y | Y | Y | Y |
| **OR** The authors described their funding sources and how they managed potential conflicts of interest |  |  |  | Y |  |  |  |  |  |  |  |  |
| *Code result: 0 = N, 1 = Y, NA* | 1 | 1 | 1 | 1 | 1 | 1 | 1 | 1 | 1 | 1 | 1 | 1 |
| **Appraisal rating** | **Critically Low** | **Critically Low** | **Low** | **Moderate** | **Critically low** | **Critically low** | **Low** | **Low** | **Low** | **Critically low** | **Low** | **Critically low** |
| Y: Yes, N: No, NA: Not applicable |  |  |  |  |  |  |  |  |  |  |  |  |
