# Supplementary material for: Mechanisms of Engagement With Mobile Health Apps for Adults With Long-Term Conditions: Overview of Systematic Reviews
Source: JMIR Mhealth Uhealth. 2026 Jul 24;14:e88382. doi: 10.2196/88382 (PMC13398183; doi:10.2196/88382)
Supplement: Multimedia Appendix 4 [file mhealth-v14-e88382-s004.docx]

| **Supplemental File 3.** Quality appraisal of qualitative and mixed-methods systematic reviews (n=7/19) using the CASP tool | | | | | | | |
| --- | --- | --- | --- | --- | --- | --- | --- |
|  | Alaslawi et al., 2022 | Bezerra Giordan et al., 2022 | Dunham et al., 2021 | O'Neill et al., 2021 | Patail et al., 2025 | Patterson et al., 2021 | Vaezipour et al., 2019 |
| 1. Was there a clear statement of the aims of the research? | yes | yes | yes | yes | yes | yes | yes |
| 2. Is a qualitative methodology appropriate? | yes | yes | yes | yes | yes | yes | yes |
| 3. Was the research design appropriate to address the aims of the research? | yes | yes | yes | yes | yes | yes | yes |
| 4. Was the recruitment strategy appropriate to the aims of the research? | NA | NA | NA | NA | NA | NA | NA |
| 5. Was the data collected in a way that addressed the research issue? | yes | yes | yes | yes | yes | yes | yes |
| 6. Has the relationship between researcher and participants been adequately considered? | Not clear | yes | yes | yes | yes | Not clear | Not clear |
| 7. Have ethical issues been taken into consideration? | NA | yes | yes | yes | yes | yes | yes |
| 8. Was the data analysis sufficiently rigorous? | yes | yes | yes | yes | yes | yes | yes |
| 9. Is there a clear statement of findings? | yes | yes | yes | yes | yes | yes | yes |
| 10. How valuable is the research? | yes | yes | yes | yes | yes | yes | yes |
| Appraisal rating: | Moderate | Moderate-to-high | Moderate | High | High | Moderate-to-high | High |
