# Supplementary material for: Mechanisms of Engagement With Mobile Health Apps for Adults With Long-Term Conditions: Overview of Systematic Reviews
Source: JMIR Mhealth Uhealth. 2026 Jul 24;14:e88382. doi: 10.2196/88382 (PMC13398183; doi:10.2196/88382)
Supplement: Multimedia Appendix 5 [file mhealth-v14-e88382-s005.docx]

| **Supplemental File 4.** mHealth app-based intervention characteristics (n=19) | | | | | | |
| --- | --- | --- | --- | --- | --- | --- |
| **Author, Year** | **App names*** | **Self-guided n (%)** | **Standalone n (%)** | **Therapeutic Use** | **App Features** | **WHO DHI Sub-category** |
| Alaslawi et al., 2022 | Health360x n=1, Apple HealthKit n=1, CONEMO n=1, unnamed=25 | 20 (71.4) | 28 (100) | Diabetes self-management and behavior change | Educational content (n=10) | 1.1.2 Transmit targeted health information to person(s) based on health status or demographics |
|  |  |  |  |  | Medication reminders (n=5), Automated feedback or decision support (n=6) | 1.1.3 Transmit targeted alerts and reminders to person(s) |
|  |  |  |  |  | Social support or peer interaction (n=2) | 1.3.1 Peer group for individuals |
|  |  |  |  |  | Blood glucose monitoring (n=15), Physical activity tracking (n=6), Weight tracking (n=3), Blood pressure tracking (n=3) | 1.4.2 Self-monitoring of health or Diagnostic data by the individual |
|  |  |  |  |  | Diet tracking/nutrition logging (n=7), Goal setting (n=4), Insulin dosage calculation (n=2), Diabetes diary or general journaling (n=4), Carbohydrate counting (n=2) | 1.4.3 Active data capture/documentation by an individual |
|  |  |  |  |  | Communication with healthcare providers (n=8) | 2.4.1 Consultations between remote person and healthcare providers |
| Bezerra Giordan et al., 2022 | Not reported | 10 (35.7) | 28 (100) | Cardiovascular self-management | Reminder Notifications for monitoring (n=12), Personalization (e.g., feedback tailored to user data) (n=9) | 1.1.3 Transmit targeted alerts and reminders to person(s) |
|  |  |  |  |  | Access to electronic medical records (n=2) | 1.4.1 Access by the individual to own medical or summary health records |
|  |  |  |  |  | Vital signs monitoring (e.g., BP, HR) (n=15), Weight monitoring (n=19), Automated monitoring via wireless devices (n=10) | 1.4.2 Self-monitoring of health or Diagnostic data by the individual |
|  |  |  |  |  | Detailed symptom tracking including severity or validated tools (n=5) | 1.4.3 Active data capture/documentation by an individual |
|  |  |  |  |  | Direct clinician communication (n=5) | 2.4.1 Consultations between remote person and healthcare providers |
| Campbell & Porter, 2015 | Diet Mate Pro, Balance Log, Dietary Intake Monitoring App | Not specified | 5 (100) | Dietary self-monitoring in dialysis patients | Dietary self-monitoring (n=5), fluid intake tracking (n=3), sodium intake tracking with visual feedback (n=4), tracking of other nutrients (potassium, phosphorus, protein, energy) (n=2), biochemical marker monitoring (e.g., serum potassium, phosphorus, albumin, CRP) (n=2), intradialytic weight gain tracking (n=3) | 1.4.2 Self-monitoring of health or Diagnostic data by the individual |
| de Melo Santana et al., 2023 | Relieve My Back n=1, Snapcare n=1, selfBACK n=1, Kaia n=1, ViViRA n=1, | 2 (40) | 5 (100) | Self-management and physical rehabilitation for low back pain | Education about low back pain (n=3) | 1.1.2 Transmit targeted health information to person(s) based on health status or demographics |
|  |  |  |  |  | Notifications/reminders to log in (n=4), Posture reminders (n=1), Walking/physical activity prompts (n=2), Sleep reminders (n=1) | 1.1.3 Transmit targeted alerts and reminders to person(s) |
|  |  |  |  |  | Physical exercises delivered via apps including stretching, strengthening or aerobic exercises (n=5), Mindfulness or relaxation techniques (n=2), goal-setting tools (n=1), pain relief exercises/tools (n=1) | 1.4.3 Active data capture/documentation by an individual |
| Diez Alvarez et al., 2024 | Pebble n=1, LG LifeGram n=1, Fitbit Alta n=1 | Not specified | 1 (33.3) | Diabetes self-management | Educational content on: Diet and/or nutrition (n=2), Medication adherence (n=1), Exercise (n=1) | 1.1.2 Transmit targeted health information to person(s) based on health status or demographics |
|  |  |  |  |  | Social support/motivation messages (n=1) | 1.1.3 Transmit targeted alerts and reminders to person(s) |
|  |  |  |  |  | Blood glucose monitoring or data display (n=4), Physical activity tracking (n=4), Diet and/or nutrition tracking and guidance (n=2), Medication adherence support and tracking (n=1) | 1.4.2 Self-monitoring of health or Diagnostic data by the individual |
|  |  |  |  |  | Reported technical issues or support needs (n=2), User interface/usability feedback collected (n=2) | 1.5.1 Reporting of health system feedback by persons |
| Dunham et al., 2021 | Keele Pain Recorder n=1, RAISE app n=2, Pathway through Pain n=1, unnamed n=6 | 8 (80) | 4 (40) | Chronic pain monitoring | Chronic pain self-management support (n=10), Patient education or pain communication support (n=6), Personalization or customization of features (n=3) | 1.1.2 Transmit targeted health information to person(s) based on health status or demographics |
|  |  |  |  |  | Clinician engagement or feedback on app use (n=6) | 1.5.1 Reporting of health system feedback by persons |
|  |  |  |  |  | Clinician engagement or feedback on app use (n=6) | 2.4.1 Consultations between remote person and healthcare providers |
| Frid et al., 2024 | SCP-A n=1, Interaktor n=1, HARUToday n=1, BPSS n=1, BCSMS n=1, mPRO Mamma n=1, BENECA n=3, Garmin wearable n=2, ASyMS n=1, unnamed n=2, Smart After-Care app n=2, CanRelax n=1, Fitbit n=1, Untire n=1, Health Mate n=1, WeChat n=1, BCS n=1, Oncokompas n=1, eRAPID n=1, AABCS n=1, WWACP n=1, LivingWith n=1, Fitbit Alta n=1, Unicare Home+ n=1, ePRO-CTCAE n=1 | Not specified | 25 (83.3) | Cancer survivorship, post-chemotherapy, side effect management | Tailored self-management information (n=8) | 1.1.2 Transmit targeted health information to person(s) based on health status or demographics |
|  |  |  |  |  | Social sharing/community features (n=3) | 1.3.1 Peer group for individuals |
|  |  |  |  |  | Physical activity tracking (n=11), Symptom tracker (n=8) | 1.4.2 Self-monitoring of health or Diagnostic data by the individual |
|  |  |  |  |  | Nutrition guidance (n=6), Activities aimed at improving mental health (n=4), Lifestyle programs (n=3) | 1.4.3 Active data capture/documentation by an individual |
|  |  |  |  |  | Expert consulting access (n=4) | 2.4.1 Consultations between remote person and healthcare providers |
| He et al., 2022 | Not reported | 2 (10.5) | 19 (100) | Diabetes self-management | Educational content (n=19), Automated feedback (n=13) | 1.1.2 Transmit targeted health information to person(s) based on health status or demographics |
|  |  |  |  |  | Symptom monitoring (n=17) | 1.4.2 Self-monitoring of health or Diagnostic data by the individual |
|  |  |  |  |  | Professional monitoring and sessions with clinicians (n=17) | 2.4.1 Consultations between remote person and healthcare providers |
| Hernandez Silva, Lawler & Langbecker, 2019 | ASyMS n=2, SurvivorCHESS n=1, Smart After Care n=1, Interaktor n=1, TOLF n=1, Cancer Distress Coach n=1 | 2 (28.6) | 7 (100) | Cancer treatment and survivorship support | Self-management information (n=6), Information about health condition (n=4), Video demonstrations (n=2), Links to online resources (n=2) | 1.1.2 Transmit targeted health information to person(s) based on health status or demographics |
|  |  |  |  |  | Reminder alerts (n=3) | 1.1.3 Transmit targeted alerts and reminders to person(s) |
|  |  |  |  |  | Networking with other users (n=1) | 1.3.1 Peer group for individuals |
|  |  |  |  |  | Progress tracking (n=5), Links with pedometer (n=2) | 1.4.2 Self-monitoring of health or Diagnostic data by the individual |
|  |  |  |  |  | Symptom questionnaire (n=6), Exercise program (n=4), survivorship care plan (n=1), Mind-body exercises (n=1) | 1.4.3 Active data capture/documentation by an individual |
|  |  |  |  |  | Connects with HCP (n=6) | 2.4.1 Consultations between remote person and healthcare provider |
| Horn et al., 2025 | Not reported | 5 (45.5) | 3 (27.3) | Psychological support | Psychoeducation (n=3), Relaxation techniques (n=1) | 1.1.2 Transmit targeted health information to person(s) based on health status or demographics |
|  |  |  |  |  | Stress management activities (n=3), symptom diary (n=1) | 1.4.2 Self-monitoring of health or Diagnostic data by the individual |
|  |  |  |  |  | Therapist support (n=6) | 2.4.1 Consultations between remote person and healthcare provider |
| Lee et al., 2022 | PDa Dr, Fox Wearable Companion app, HopkinsPD, PD Manager, eDiary app, 9zest Parkinson’s Therapy, EncephaLog Home, SymTrend, Beats Medical Parkinson’s Treatment App, STOP (the Sentient Tracking of Parkinson’s) app, CuPiD system, PTA (the Parkinson’s Tracker App), Wellpepper | 4 (23.6) | 10 (58.8) | Parkinson’s Disease (PD) management | Reminder and user interaction functions (n=17) | 1.1.3 Transmit targeted alerts and reminders to person(s) |
|  |  |  |  |  | Patient Outcomes (n=5) | 1.4.2 Self-monitoring of health or Diagnostic data by the individual |
|  |  |  |  |  | Symptom Data Collection (n=17) | 1.4.3 Active data capture/documentation by an individual |
| MacLean et al., 2025 | Not reported | 8 (100) | 8 (100) | Chronic pain self-management | Relaxation techniques (n=4) | 1.1.2 Transmit targeted health information to person(s) based on health status or demographics |
|  |  |  |  |  | Reminders (n=3) | 1.1.3 Transmit targeted alerts and reminders to person(s) |
|  |  |  |  |  | Diaries (n=3), gamification (n=2), self-monitoring (n=2), exercise videos (n=3) | 1.4.2 Self-monitoring of health or Diagnostic data by the individual |
| Magalhães et al., 2021 | MPP, ASyMS, Pit-a-Pat, Consilium, Life Manager, BCS, Smartphone Aftercare | 0 (0) | 9 (90) | Cancer treatment toxicity monitoring & symptom self-management | Educational content (n=6) | 1.1.2 Transmit targeted health information to person(s) based on health status or demographics |
|  |  |  |  |  | Alerts and Notifications (n=5) | 1.1.3 Transmit targeted alerts and reminders to person(s) |
|  |  |  |  |  | Symptom monitoring (n=9), Passive data collection (n=2) | 1.4.2 Self-monitoring of health or Diagnostic data by the individual |
| O'Neill et al., 2021 | PilAm Go4Health n=1, Fitbit diary app n=1, MoDD n=1, Few touch n=1, Unnamed n=10 | 14 (100) | 12 (85.7) | Diabetes self-management | Educational content (n=13) | 1.1.2 Transmit targeted health information to person(s) based on health status or demographics |
|  |  |  |  |  | Reminders (n=7), Personalized feedback (n=8) | 1.1.3 Transmit targeted alerts and reminders to person(s) |
|  |  |  |  |  | Social support (n=13) | 1.3.1 Peer group for individuals: |
|  |  |  |  |  | Data tracking (n=14) | 1.4.2 Self-monitoring of health or Diagnostic data by the individual |
|  |  |  |  |  | Goal setting (n=13), Problem solving activities (n=1) | 1.4.3 Active data capture/documentation by an individual |
| Patail et al., 2025 | Not reported | Not reported | Not reported | Diabetes self-management | Interrelationship Knowledge (glucose/diet/exercise/medication) (n=17) | 1.1.2 Transmit targeted health information to person(s) based on health status or demographics |
|  |  |  |  |  | Reminder features (glucose checks, medication, appointments, meals) (n=8) | 1.1.3 Transmit targeted alerts and reminders to person(s) |
|  |  |  |  |  | Peer support/chat features (n=8) | 1.3.1 Peer group for individuals: |
|  |  |  |  |  | Blood glucose, diet, exercise monitoring (n=20), Personalized diabetes type-specific features (n=4) | 1.4.2 Self-monitoring of health or Diagnostic data by the individual |
|  |  |  |  |  | Real-time HCP communication (n=1) | 2.4.1 Consultations between remote person and healthcare provider |
| Patterson et al., 2021 | Vida Health + Fitbit n=1, Fitbit 1, Vire =1, Fitlab n=1, SUPPORT n=1, HealthyCircles n=1, Vett n=1, EMPOWER-H n=1, STARFISH n=1, Farmalarm n=1, HeartCycle GEx n=1, HerBEAT n=1, MEMRS-CRS+We Chat n=1, DASH n=1, CPMP n=1, Personal Health Assistant n=2 | Not specified | 12 (63.2) | Improving physical activity in people with CVDs | Educational Content (n=19), Personalized module order (n=16) | 1.1.2 Transmit targeted health information to person(s) based on health status or demographics |
|  |  |  |  |  | Activity tracking (n=7), Symptom monitoring (n=9) | 1.4.2 Self-monitoring of health or Diagnostic data by the individual |
|  |  |  |  |  | Self-report measures (n=12) | 1.4.3 Active data capture/documentation by an individual |
| Rintala et al., 2023 | Android app n=1, FINDEX n=1, MoU-Rehab n=1, Fitbit Training and Fitlab Test n=1, Finger training application n=1, Tap-it n=1, ARMStrokes n=1, STARFISH n=1, Farmalarm n=1, 9zest Stroke  Rehab App n=1, CARE4STROKE n=1 | 9 (81.8) | 10 (90.9) | Post-stroke rehabilitation, motor recovery, and balance training | Exercise prescription (n=3), Personalization (n=6) | 1.1.2 Transmit targeted health information to person(s) based on health status or demographics |
|  |  |  |  |  | Gaming (n=6), Monitoring (n=2) | 1.4.2 Self-monitoring of health or Diagnostic data by the individual |
| Vaezipour et al., 2019 | SAMI n=1, Unnamed n=3 | 4 (100) | 4 (100) | Improving cognitive impairment and daily functioning in people with TBI | Supported planning/money management (n=3), Cognitive functioning support (n=3) | 1.1.2 Transmit targeted health information to person(s) based on health status or demographics |
|  |  |  |  |  | Reminders (n=2) | 1.1.3 Transmit targeted alerts and reminders to person(s) |
|  |  |  |  |  | Monitoring (n=4) | 1.4.2 Self-monitoring of health or Diagnostic data by the individual |
|  |  |  |  |  | Appointment with healthcare providers (n=1) | 2.4.1 Consultations between remote person and healthcare provider |
| Whitehead & Seaton, 2016 | Diabeo, Few Touch Application, Glucose Buddy, Java 2 Micro Edition app, unnamed, t+ Asthma, DialBetics | 2 (22.3) | 3 (33.3) | Symptom management | Reminders (n=6) | 1.1.2 Transmit targeted health information to person(s) based on health status or demographics |
|  |  |  |  |  | Automated messages/Notifications (n=7) | 1.1.3 Transmit targeted alerts and reminders to person(s) |
|  |  |  |  |  | Data tracking (n=8) | 1.4.2 Self-monitoring of health or Diagnostic data by the individual |
|  |  |  |  |  | Clinician feedback (n=7) | 2.4.1 Consultations between remote person and healthcare provider |
| *Where reported, provide the number of mHealth app-based interventions in the systematic reviews. |  |  |  |  |  |  |
