# Supplementary material for: Mechanisms of Engagement With Mobile Health Apps for Adults With Long-Term Conditions: Overview of Systematic Reviews
Source: JMIR Mhealth Uhealth. 2026 Jul 24;14:e88382. doi: 10.2196/88382 (PMC13398183; doi:10.2196/88382)
Supplement: Multimedia Appendix 6 [file mhealth-v14-e88382-s006.docx]

| **Supplemental File 5.** Descriptive engagement indicators and their impact on health outcomes (n=19) | | | | |  |
| --- | --- | --- | --- | --- | --- |
| **Author, year** | **A priori definition** | **Primary studies (n)** | **Descriptive indicators** | **Impact on health outcomes** | |
| Alaslawi et al., 2022 | Factors affecting patients’ use of DSM apps | Not reported | - |  | |
| Bezerra Giordan et al., 2022 | Not defined | 3 | App usage: <50% used daily; 60% used more than once/week. | App use (≥50% engagement) linked to improved heart failure knowledge and quality of life. | |
| Campbell & Porter, 2015 | Not defined, used synonymously with usage and engagement | 4 | Usage ranged from <50% of available days to >80%; 1.9–3.05 meals logged/day. | ≥50% engagement linked to reduced sodium intake | |
| de Melo Santana et al., 2023 | Different definitions in primary studies, synthesized narratively as 'adherence' in the review, but there was no definition for 'adherence' | 4 | Daily app use 6× higher than control; average usage of 35 days over 12 weeks; 78% adherence. | No significant relationship between adherence and symptom improvements. | |
| Diez Alvarez et al., 2024 | Not defined, primary studies synthesized narratively in the review which included engagement indicators | 3 | 87.5% of daily PA submissions completed; other indicators (e.g., glucose, adherence) ranged from 54–71%. | Higher input scores associated with HbA1c improvement. | |
| Dunham et al., 2021 | Elements of self-care support for chronic pain that can be delivered by via smartphone interventions | Not reported | - | - | |
| Frid et al., 2024 | Not defined, synthesized primary studies narratively as 'adherence' in the review | 5 | 59.6% used app over 12 weeks; mean use: 18 days, 13.4 mins/day; adherence dropped from 100% to 74.5%; stable step count (~7,000). | Consistent adherence (>6 days/week) linked to increased physical activity levels. | |
| He et al., 2022 | Different definitions in primary studies, synthesized narratively as 'engagement' in the review | 5 | Logged use avg. 42.4 days over 26 weeks; 18.2% logged >100 days; ⅓ classified as high users. | - | |
| Hernandez Silva, Lawler & Langbecker, 2019 | Not defined, synthesized as 'intervention uptake and use' and 'user perceptions' in the review | 7 | 94–100% initial uptake; >90% continued use; declining use over time; most-used features: tracking, networking. | - | |
| Horn et al., 2025 | Not defined | 11 | Mean usage = 25.7 days (SD = 33.9) over 3 months; median=63 min/week for 1.5 months. Logins ranged from 0–45; total duration=0–2324 mins. | Higher engagement linked to improved self-efficacy; reduced distress, depression, anxiety and fear of recurrence. | |
| Lee et al., 2022 | Not defined, synthesized as 'compliance' in the review | 7 | Compliance ranged: high (87–96%) to moderate (43–67%); one study reported 29.6%. | - | |
| MacLean et al., 2025 | System use | 7 | Completion: 3 of 12 modules; Dropout rates: 16–71%; App use declined from 84% to 29% over 12 weeks | Higher engagement associated with lower pain. | |
| Magalhães et al., 2021 | Not defined, synthesized narratively | 8 | Five studies reported adherence ranging from 20% to 84.8%, with engagement measures varying widely (e.g., number of logged activities, conversations with the app), making comparisons challenging. Dropout rates ranged from 0% to 87%. | Higher engagement linked to better self-efficacy, quality of life, physical strength, social support; reduced fatigue, nausea. | |
| O'Neill et al., 2021 | The experiences of using mobile health (mHealth) applications | Not reported | - |  | |
| Patail et al., 2025 | UTAUT framework | Not reported | - | - | |
| Patterson et al., 2021 | Not defined, synthesized as 'intervention uptake, engagement, adherence and dropout' in the review | 19 | Adherence: 20–84.8%; engagement measures varied widely; dropout: 0–87%. | - | |
| Rintala et al., 2023 | Not defined | 4 | Mixed adherence: 5.7 sessions/week; 50% adherence to walking; 14% for core stability; 75% completed tapping task. | - | |
| Vaezipour et al., 2019 | Part of user acceptance categories | Not reported | - | - | |
| Whitehead & Seaton, 2016 | Not defined, synthesized as usability, feasibility and acceptability in the review | 7 | Attrition: 8.75–26%; adherence: 71.7–80% (intervention), 76.7–46.7% (control); daily data entry time: 10s–22.5m; module compliance: 50–70%. |  | |
