# Supplementary material for: Mechanisms of Engagement With Mobile Health Apps for Adults With Long-Term Conditions: Overview of Systematic Reviews
Source: JMIR Mhealth Uhealth. 2026 Jul 24;14:e88382. doi: 10.2196/88382 (PMC13398183; doi:10.2196/88382)
Supplement: Multimedia Appendix 7 [file mhealth-v14-e88382-s007.docx]

| **Supplemental File 6.** Mapping modifiable barriers and facilitators of engagement to the Self-Determination Theory (SDT) | | | | | | |
| --- | --- | --- | --- | --- | --- | --- |
| **Theme** | **Sub-theme** | **Example quote** | **Category** | **SLRs n=** | **Total primary studies n=** | **SDT** |
| App design | Design accessibility | “Small font size, touch screen difficulties, switching between numbers and letters, and content navigation difficulties.” | Barriers | 2 | 7 | C |
|  | Monotonous design | “…reasons for dropping out included… app too monotonous…” | Barriers | 1 | 1 | R |
|  | Tech problems with the app | “Challenges such as frequent crashing of apps, slow downloading of information, incompatible monitoring devices and Bluetooth issues.”  “The lack of automated capability for uploading information made the task… overwhelming and time-consuming.” | Barriers | 7 | 41 | A & C |
|  | Ease of use | "Patients are more likely to use DSM apps if they are easy to use" | Facilitator | 4 | 7 | C |
|  | Language | Participants noted the “...importance of using plain English and having clear and accessible instructions.” | Facilitator | 2 | 10 | C |
|  | Visual aids | “The colorful graphs depicting interaction of diet and exercise with glucose levels were perceived to be educational and confidence enhancing.” | Facilitator | 4 | 39 | C |
| Baseline health behavior change readiness | Perceived need | “No, I don’t need an app, I think I’m keeping it well under control.” | Barriers | 2 | 16 | A |
|  | Psychological readiness | “There’s that whole accountability piece… If I’m doing this, then I’m going to have to pay more attention.” | Barriers | 3 | 10 | A |
|  | Thoughts of potential harm to LTC | “Some expressed apprehension related to the potential for amplification of anxiety, negative emotions and catastrophizing.” | Barriers | 2 | 3 | C |
|  | Curiosity | “[I] am trying to educate myself… that is why I liked these tools.” | Facilitator | 1 | 4 | A & C |
| Baseline technological readiness | Digital literacy/Complexity of tech | "If you’re not very tech savvy, which I am not, I don’t care how easy it is. It’s not always that easy’’ | Barrier and facilitator | 4 | 26 | C |
|  | App likeability | “Some stopped using apps because they didn’t like the device.” | Barriers | 1 | 1 | R |
|  | Availability of tech support | “Need to increase the level of supports for downloading and setting up the app… due to low confidence with technology.”  “Participants… found the application easy to use and helpful in assisting them to manage their daily life activities.” | Barrier and facilitator | 4 | 10 | C & R |
| Time burden (Ease of integrating to daily life) |  | "Patients were less likely to use DSM apps if they could not integrate the app with daily activities, creating time constraints" | Barriers | 3 | 10 | A |
| Trust | Data privacy | “Participants… were concerned about data privacy and how their data might be used and safeguarded.”  "Patients are more likely to use DSM apps if they ensure data privacy and security" | Barrier and facilitator | 2 | 11 | A |
|  | HCP reinforcement | “Participants were uncertain whether the information provided was validated by medical doctors.”  “Participants would not use the apps unless recommended by their attending physicians.” | Barrier and facilitator | 1 | 13 | R |
| App functionality | Data entry burden | "participants dropped out specifically because ...the frequency of input was too burdensome.” | Barrier | 2 | 10 | A & C |
|  | Did not meet expectation | “I wish there was something that could give me advice about nutrition… it didn’t do that at all.” | Barrier | 1 | 5 | C |
|  | Interaction with other users |  | Barrier and facilitator | 5 | 23 | R |
|  | Data sharing with people for emergencies | "Sharing data easily with clinicians, family, and caregivers during emergencies was commonly considered advantageous" | Facilitator | 2 | 18 | R |
|  | Interaction with HCPs | “They can reach out to you… let’s schedule a visit earlier so I can help get you back on track.” | Facilitator | 5 | 29 | R |
|  | Goal setting | patients are more likely to use DSM apps if they set up goals | Facilitator | 2 | 7 | A |
|  | Integration and access to medical records | Participants in several studies considered that the app could be an excellent tool... particularly if it allowed for data sharing and integration with electronic medical records | Facilitator | 2 | 9 | R |
|  | Personalized and relatable information | "Noted the need for personalization of the intervention and content provided and their preference for more personalization in the ability to report symptoms and needs, which ideally would also generate more relevant feedback"  “Participants expressed interest in apps with features tailoring to the different types of diabetes.” | Facilitator | 5 | 15 | A & C |
|  | Psychoeducation | “Information overload was noted as the most important barrier to device implementation.”  “Participants [with diabetes] valued the apps as a source of information on the effect of diet, exercise and medication on glucose levels.” | Barrier and facilitator | 3 | 28 | C |
|  | Real-time feedback | patients are more likely to use DSM apps if they get real-time feedback | Facilitator | 2 | 12 | C |
|  | Frequency of reminders | “Participants reported that audible and visual reminders were useful in assisting them to remember daily tasks and appointments.” | Facilitator | 4 | 15 | A |
|  | Tone of reminders | “Tone of the reminder alerts were perceived to be annoying and generated stigmatization.” | Barrier | 1 | 2 | R |
|  | Symptom monitoring | While you may have an idea of how you are managing your diabetes, the graphical information presented in the app clearly shows how well you are in fact managing.  Most study participants appreciated and noted the importance of automated self-monitoring (particularly through wireless device integration | Facilitator | 3 | 17 | A + C |
| Awareness of app |  | “95% were unaware of such applications.” | Barriers | 2 | 6 | External motivation |
| Availability of digital tool |  | “Limited availability or inconsistency of mobile phone signal… in rural areas.” | Barriers | 3 | 6 | External motivation |
| Cost |  | “They should be free… it can be life and death… people can’t afford to access full features.”  “Support for costs… via health insurance schemes or subsidy from government… was perceived to facilitate uptake.” | Barrier and facilitator | 4 | 17 | External motivation |
| A= Autonomy, C=Competence, R=Relatedness. | | | | | | |
