# Supplementary figures and images for: Mechanisms of Engagement With Mobile Health Apps for Adults With Long-Term Conditions: Overview of Systematic Reviews
Source: JMIR Mhealth Uhealth. 2026 Jul 24;14:e88382. doi: 10.2196/88382 (PMC13398183; doi:10.2196/88382)

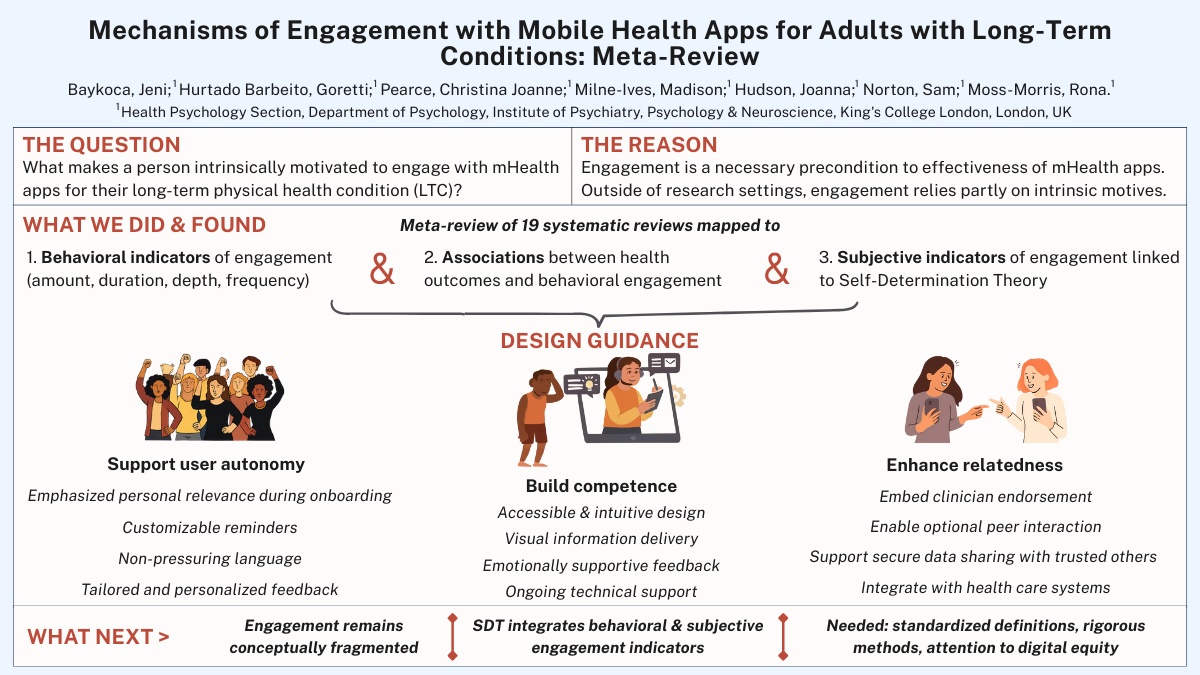

Supplement: Multimedia Appendix 8 [file mhealth-v14-e88382-s008.png]
